# Statistical analysis of hard X-ray radiation at the PAL-XFEL facility performed by Hanbury Brown and Twiss interferometry

**DOI:** 10.1107/S1600577522008773

**Published:** 2022-10-07

**Authors:** Young Yong Kim, Ruslan Khubbutdinov, Jerome Carnis, Sangsoo Kim, Daewoong Nam, Inhyuk Nam, Gyujin Kim, Chi Hyun Shim, Haeryong Yang, Myunghoon Cho, Chang-Ki Min, Changbum Kim, Heung-Sik Kang, Ivan A. Vartanyants

**Affiliations:** a Photon Science, Deutsche Elektronen-Synchrotron DESY, Notkestrasse 85, 22607 Hamburg, Germany; b Pohang Accelerator Laboratory, Pohang, Gyeongbuk 37673, Republic of Korea; c Photon Science Center, POSTECH, Pohang 37673, Republic of Korea; RIKEN SPring-8 Center, Japan

**Keywords:** Hanbury Brown and Twiss interferometry, second-order correlation functions, X-ray free-electron lasers, statistical properties

## Abstract

A Hanbury Brown and Twiss interferometry experiment based on second-order correlations was performed at the PAL-XFEL facility. The statistical properties of the X-ray radiation were studied within this experiment. Measurements were performed at the NCI beamline at 10 keV photon energy under various operation conditions: self-amplified spontaneous emission (SASE), SASE with a monochromator, and self-seeding regimes at 120 pC, 180 pC and 200 pC electron bunch charge. Statistical analysis showed short average pulse duration from 6 fs to 9 fs depending on the operational conditions. A high spatial degree of coherence of about 70–80% was determined in the spatial domain for the SASE beams with the monochromator and self-seeding regime of operation. The obtained values describe the statistical properties of the beams generated at the PAL-XFEL facility.

## Abstract 2

Statistical properties of the hard X-ray free-electron laser PAL-XFEL were studied by Hanbury Brown and Twiss interferometry. The results demonstrate high spatial coherence and short average pulse duration of this facility at 10 keV photon energy.

## 1. Introduction

Hard X-ray free-electron lasers (XFELs) are currently the brightest X-ray sources in the world (Emma *et al.*, 2010 ▸; Ishikawa *et al.*, 2012 ▸; Kang *et al.*, 2017 ▸; Decking *et al.*, 2020 ▸; Prat *et al.*, 2020 ▸). These facilities provide intense hard X-ray beams with high coherence properties and pulse durations in the range of tens to hundreds of femtoseconds (McNeil & Thompson, 2010 ▸; Pellegrini *et al.*, 2016 ▸). Such unique properties have triggered research in atomic physics (Young *et al.*, 2010 ▸; Rohringer *et al.*, 2012 ▸; Prince *et al.*, 2016 ▸), structural dynamics that determine the function of proteins (Kern *et al.*, 2013 ▸; Nogly *et al.*, 2018 ▸), mechanisms controlling chemical bonds during catalytic reactions (Dell’Angela *et al.*, 2013 ▸; Öström *et al.*, 2015 ▸), and the processes that are interesting for energy conversion and information storage applications (Beaud *et al.*, 2014 ▸; Dornes *et al.*, 2019 ▸). The high peak intensities and short pulse duration generated by these facilities have introduced entirely new fields of research such as femtosecond crystallography (Chapman *et al.*, 2011 ▸) and single-particle imaging (SPI) (Seibert *et al.*, 2011 ▸; Aquila *et al.*, 2015 ▸), allowing the determination of a three-dimensional biological particle with a resolution of less than 10 nm (Rose *et al.*, 2018 ▸; Assalauova *et al.*, 2020 ▸). Furthermore, the advantages of XFELs have allowed such coincidence-based experiments as incoherent and ghost imaging to be performed (Schneider *et al.*, 2018 ▸; Kim *et al.*, 2020 ▸).

A crucial factor in generating the unique characteristics of XFEL radiation is the X-ray lasing process. The key principle utilized at most of the XFEL facilities is based on the self-amplified spontaneous emission (SASE) process, allowing the generation of highly intense XFEL pulses (Saldin *et al.*, 2000 ▸; Milton *et al.*, 2001 ▸). The beams generated by SASE radiation have a high degree of spatial coherence and many longitudinal modes that vary randomly from one pulse to another. It was demonstrated experimentally that statistically such XFELs behave as a chaotic source^1^ (Singer *et al.*, 2013 ▸; Song *et al.*, 2014 ▸; Gorobtsov *et al.*, 2017 ▸, 2018*a* ▸; Khubbutdinov *et al.*, 2021 ▸). An important exception to this rule is the externally seeded FEL at the FERMI facility in Trieste (Italy), which behaves as a truly one-mode laser source with a high degree of spatial coherence (Gorobtsov *et al.*, 2018*a* ▸).

As was demonstrated at the Linac Coherent Light Source (LCLS), one of the ways to obtain a narrow spectral line about the size of a single spike in the SASE spectrum is to perform self-seeding (Amann *et al.*, 2012 ▸). Self-seeding at hard XFELs is based on installing a diamond crystal of high quality in Bragg geometry instead of the undulator section in the undulator line (Geloni *et al.*, 2011 ▸). After successful commissioning of hard X-ray radiation generated by SASE at the Pohang Accelerator Laboratory X-ray Free-Electron Laser (PAL-XFEL) in 2016 (Kang *et al.*, 2017 ▸), the self-seeding operation was also successfully implemented in 2018 (Min *et al.*, 2019 ▸; Nam *et al.*, 2021 ▸). Now, a natural question is: what are the statistical properties of the self-seeded X-ray beams from the XFEL sources? Are they laser-like as in the case of externally seeded FELs (Allaria *et al.*, 2013 ▸) or do they have a rather chaotic nature like in SASE FELs?

In order to answer these fundamental questions about the statistical properties of self-seeded XFELs, one may use the method of Hanbury Brown and Twiss (HBT) interferometry. The method is based on second-order intensity correlations and was first introduced experimentally by Hanbury Brown and Twiss (Hanbury Brown & Twiss, 1956 ▸; Brown & Twiss, 1956 ▸). Later, it led to the creation and development of the field of quantum optics (Glauber, 1963 ▸; Sudarshan, 1963 ▸). Currently, this method has been successfully applied for analysis of X-ray radiation at different FEL facilities (Singer *et al.*, 2013 ▸; Gorobtsov *et al.*, 2017 ▸, 2018*b* ▸; Inoue *et al.*, 2018 ▸; Khubbutdinov *et al.*, 2021 ▸).

In this work, we present a statistical analysis of the hard X-ray beams generated by PAL-XFEL under different operation conditions using HBT interferometry. These conditions are: SASE radiation, SASE radiation with a monochromator, and self-seeding regime of operation. The latter is of particular interest in terms of understanding the self-seeding operational mode of this facility.

## 2. HBT interferometry

HBT interferometry is a method that uses the second-order correlation of intensity measured in spatial or temporal domains and is effective in analysing the statistical properties of optical wavefields. The normalized second-order correlation function in the spatial domain is expressed as

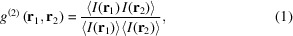

where *I*(**r**_1_) and *I*(**r**_2_) are the intensities of the wavefield in the spatial domain, and averaging, denoted by angular brackets 〈…〉, is performed over a large ensemble of different realizations of the wavefield. A similar expression will hold in the spectral domain.

If radiation is cross-spectrally pure and obeys Gaussian statistics, which means it is analogous to a chaotic source (Mandel & Wolf, 1995 ▸), the *g* ^(2)^ function may be expressed as (Ikonen, 1992 ▸; Singer *et al.*, 2013 ▸; Vartanyants & Khubbutdinov, 2021 ▸)

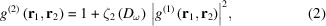

where *g* ^(1)^(**r**_1_, **r**_2_) = *E* ^*^(**r**_1_)*E*(**r**_2_)/[*I*(**r**_1_)*I*(**r**_2_)]^1/2^ is the first-order correlation function or spectral degree of coherence and 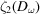 is the contrast function, which depends on the radiation bandwidth *D*_ω_. The contrast, 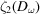 , is proportional to τ_c_/*T* in the limit when the average pulse duration (*T*) is much larger than the coherence time (τ_c_) (*T* ≫ τ_c_). Conversely, if the coherence time is larger than the pulse duration, the contrast has a constant value close to 1 (Singer *et al.*, 2013 ▸; Vartanyants & Khubbutdinov, 2021 ▸).

For the XFEL sources the *g* ^(2)^ function could be affected by fluctuations and instabilities of the machine and downstream optics. We discuss this in detail in Section 4 by providing theoretical simulations of different instabilities that were also observed in our analysis.

## 3. Results

### 3.1. Experiment

**Figure 1:**
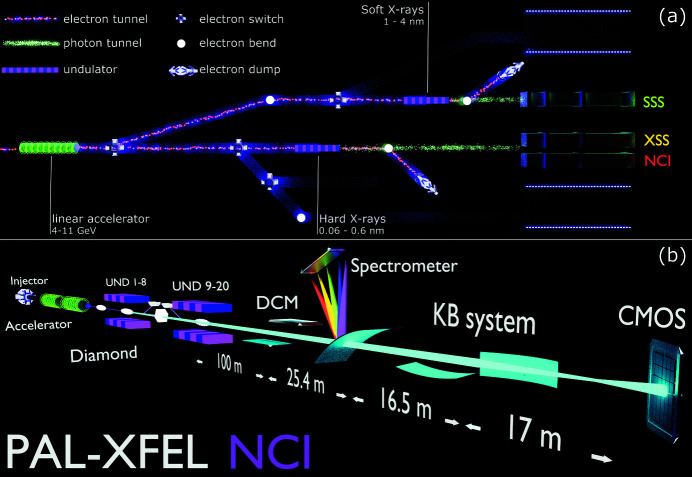
Schematic image of the experimental set-up. (*a*) The general outline of the PAL-XFEL facility. (*b*) An outline of the NCI beamline at PAL-XFEL. For the SASE radiation, 20 undulator sections were used. In the self-seeding mode of operation, the diamond crystal between the eighth and ninth sections of the undulator was implemented.

**Figure 2:**
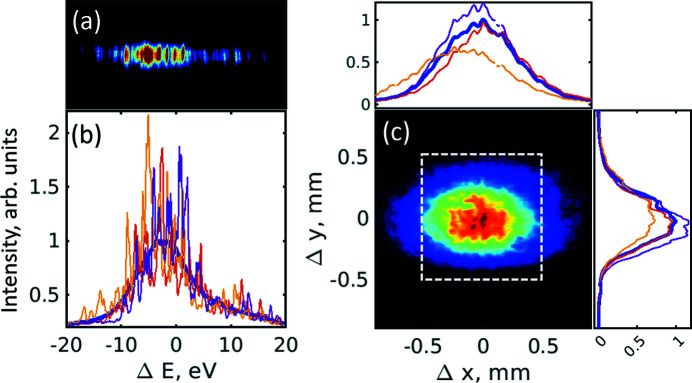
Spectral and spatial intensity distributions for the SASE operating conditions with the 180 pC bunch charge. (*a*) Typical 2D spectral intensity distribution of a single pulse in the SASE mode measured by the Andor CCD detector after subtraction of the dark images. (*b*) Projection of several individual pulses in the vertical direction and an average spectrum (blue line). (*c*) Average spatial intensity distribution in the SASE mode measured by the Hamamatsu detector. The region of interest for the correlation analysis in the spatial domain is shown in (*c*) by the white dashed square. A projection of individual pulses as well as an average intensity distribution (blue curve) in the horizontal and vertical directions is also shown in panel (*c*).

The HBT experiment was performed at the Nano-crystallography and Coherent Imaging (NCI) hard X-ray beamline at PAL-XFEL (Park *et al.*, 2016 ▸; Kang *et al.*, 2017 ▸). PAL-XFEL was operated at 10 GeV electron energy with three different electron bunch charges of 120 pC, 180 pC and 200 pC, with 30 Hz repetition rate. A schematic image of the experimental set-up is shown in Fig. 1 ▸. The X-ray photon energy for the experiment was 10 keV (λ = 1.24 Å) with 20 sections of undulators which were 5 m in length in the saturation regime (Ko *et al.*, 2017 ▸). For all bunch charges the experiment was performed with SASE radiation, SASE radiation with the monochromator, and self-seeded radiation modes. In addition to these modes, a linear regime was used in a few cases with 12 undulator sections for the 120 pC bunch charge and 13 undulator sections for the 200 pC bunch charge. Typical recorded data for SASE single pulses are shown in Fig. 2 ▸ for the 180 pC bunch charge and in Fig. S1 of the supporting information (SI) for the 120 pC bunch charge.

For the monochromatic operation, a double-crystal Si (111) monochromator (DCM) was installed, positioned 99.84 m downstream from the source point. The theoretical resolution of the DCM was Δ*E*/*E* = 1.865 × 10^−4^ at 10 keV photon energy (X-ray server, https://x-server.gmca.aps.anl.gov). During the analysis of our experiment we observed vertical position drifts of the monochromator that were corrected by further analysis (see SI Fig. S2).

For the self-seeded operation, the forward Bragg diffraction (FBD) diamond monochromator was used, which was located after eight undulators and amplified with 12 undulators downstream (Min *et al.*, 2019 ▸; Nam *et al.*, 2021 ▸).

The spectrum of each pulse was measured by an on-line spectrometer. The spectrometer consists of a Si (333) bent crystal and an Andor detector^2^ (ZYLA5.5X-FO, 2560 × 400 pixels, pixel size 6.5 µm × 6.5 µm) positioned at 1.17 m from the bent silicon crystal (Ko *et al.*, 2017 ▸). The dispersion value at the position of the spectrometer detector was estimated to be 6 eV mm^−1^. The resolution of the on-line spectrometer was estimated to be 0.26 eV (FWHM) (Nam *et al.*, 2021 ▸). The on-line spectrometer was located 25.4 m downstream from the DCM.

All spatial measurements were performed with the focused beam using Kirkpatrick–Baez (KB) mirrors located 5.37 m upstream of the focal position. The spatial beam intensities were measured by a Hamamatsu X-ray sCMOS camera^3^ (model C12849-U101U, 2048 × 2048 pixels, pixels size 6.5 µm × 6.5 µm). The region of interest, where data were collected during the experiment, was defined as 600 × 600 pixels. This detector was positioned 11.5 m downstream from the focal position. To prevent beam damage of the spectral and spatial detectors, a 0.28 mm-thick silicon attenuator was positioned in front of the Andor detector, and silicon attenuators of different thicknesses from 1.175 mm to 1.5 mm, depending on the beam conditions, were positioned in front of the Hamamatsu detector.

### 3.2. Spectral analysis

**Figure 3:**
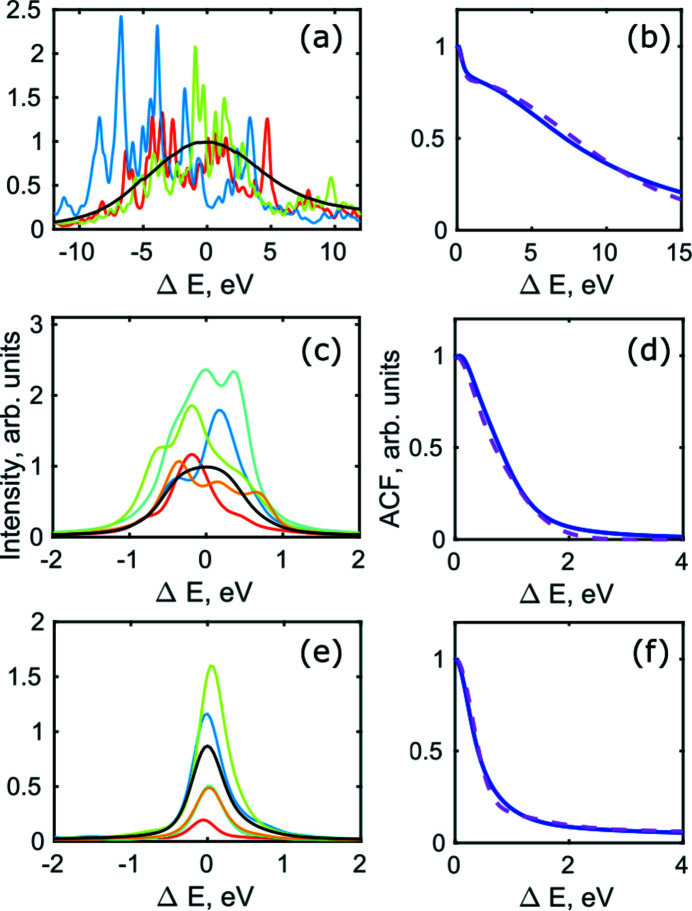
(*a*, *c*, *e*) Spectral distribution of a few random pulses and an average spectrum for all pulses (black lines). (*b*, *d*, *f*) Average autocorrelation function of all spectral lines (full lines), and its fit (dashed lines) by two Gaussian functions. (*a*, *b*) SASE radiation, (*c*, *d*) monochromatic radiation, (*e*, *f*) self-seeding regime of operation. All results presented in this figure correspond to the 180 pC bunch charge.

Single-pulse spectra and intensities in the spatial domain were collected simultaneously. To obtain statistically relevant results we collected from 8000 to 20000 pulses (see SI Table S1) at each operating condition of PAL-XFEL. Each spectrum and intensity in the spatial domain was corrected by a mean dark image with 1000 shots. The one-dimensional single-pulse spectrum was obtained by projection of the two-dimensional spectrum image along the vertical direction (see Fig. 2 ▸ and SI Fig. S1). From that, we obtained the single-pulse spectral intensity distribution, as well as an average spectrum for all operating conditions [see Figs. 3 ▸(*a*), 3(*c*) and 3(*e*) for the 180 pC bunch charge and SI Figs. S3 and S4 for the other bunch charges].

From the average spectrum we estimated the full width at half-maximum (FWHM) of the spectrum for all operation conditions (see Table 1 ▸). We observed that the width of the SASE spectrum was about 12 eV for the 120 pC and 180 pC bunch charges and is close to a single Gaussian [this was similar to the SASE operation described by Min *et al.* (2019 ▸) and Nam *et al.* (2021 ▸)]. Contrary to that, the averaged SASE spectrum for the 200 pC bunch charge was more than twice as wide (∼28 eV) and may be well represented by a sum of two Gaussian functions shifted in energy. Such different spectral behaviour at different bunch charges strongly depends on the specific machine tuning by the XFEL operators. At the same time, for all three operation conditions, the monochromatic radiation has the same bandwidth of about 1.1–1.2 eV. This value is slightly narrower than that provided by the theoretical bandwidth of the DCM at 10 keV (*ΔE* = 1.9 eV). The reason for this may be a slight detuning of two Si crystals from which the DCM is composed. For the self-seeding regime of operation, we also observed the same behaviour for all three bunch charges – the average spectrum was extremely narrow and was about 0.4 eV (see Table 1 ▸).

From the average spectrum we can determine the coherence time of the PAL-XFEL radiation for different operation conditions. The coherence time is given by the following expression (Goodman, 2000 ▸; Mandel & Wolf, 1995 ▸; Khubbutdinov *et al.*, 2021 ▸),

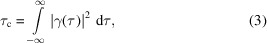

where γ(τ) is the complex degree of coherence. The complex degree of coherence may be determined through the average spectrum *S*(ω) as

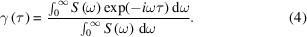

For simple spectral shapes, the coherence time has been determined by Goodman (2000 ▸). If the spectral shape is given by a Gaussian function, then 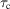 = 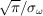 , where σ_ω_ is the root mean square (r.m.s.) value of the Gaussian spectrum. Since the average spectrum in our experiment does not follow the shape of a single Gaussian function, we used the sum of two Gaussian functions to obtain an estimate of the coherence time of the radiation in all operating conditions (see SI Table S2 for parameters of these Gaussian functions). The coherence time for a spectrum modelled by a sum of two Gaussian functions may be expressed as (Khubbutdinov *et al.*, 2021 ▸)

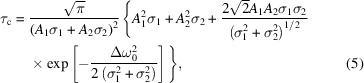

where *A*_1_ and *A*_2_ are scale factors, 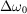 = 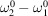 , 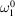 and 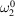 are the centres of each Gaussian line, and σ_1_ and σ_2_ are their r.m.s. values. The results of fitting the average spectrum by two Gaussian functions for all operation conditions are shown in SI Fig. S5. The determined values of the coherence time according to equation (5) are summarized in Table 1 ▸.

The values of coherence time were about 170 as for the 120 pC and 180 pC bunch charges which are typical for hard X-ray SASE operation at different XFEL facilities (Vartanyants *et al.*, 2011 ▸; Gutt *et al.*, 2012 ▸; Lehmkühler *et al.*, 2014 ▸). For monochromatic radiation, coherence times increased up to 2.5 fs for both bunch charges. Due to a broader spectrum in the case of the 200 pC bunch charge, the coherence times for SASE radiation were about twice as short and about 110 as. Interestingly, for monochromatic radiation with the 200 pC bunch charge, coherence times were 2.2 fs, similar to other bunch charges. The latter is explained by the fact that the bandwidth of monochromatic radiation is given by the DCM, which was the same for all three bunch charges. In the case of self-seeding operation mode, the sharp spectrum was staying on a broad pedestal. This pedestal originates from longitudinal phase space modulations produced by the microbunching instability upstream of the undulators as well as the SASE background (Nam *et al.*, 2021 ▸). In our estimates of the coherence time we used this sharp peak above the broad background which gave us, finally, about twice as long coherence times (∼4 fs) in comparison with monochromatic radiation for all bunch charges.

Analysis of the averaged auto-correlation function (ACF) allowed us to determine the bandwidth of a single spike in single pulse spectra (Khubbutdinov *et al.*, 2021 ▸). The ACF in Fig. 3 ▸ were fitted by a sum of two Gaussian functions as

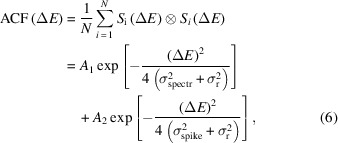

where *S*_i_(Δ*E*) is the individual spectral line measured by the on-line spectrometer for each pulse, *N* is the number of pulses, 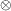 is a correlation sign, *A*_1_ and *A*_2_ are normalization constants, σ_spectr_ is the r.m.s. value of an averaged spectrum, σ_spike_ is the r.m.s. value of an average spike, and σ_r_ is the r.m.s. value of the resolution of the on-line spectrometer that was considered to be σ_r_ = 0.11 eV (Nam *et al.*, 2021 ▸). We performed the ACF analysis for all three bunch charges and operation conditions studied at PAL-XFEL and observed similar profiles for each operation mode regardless of the bunch charge (see Fig. 3 ▸ for the 180 pC bunch charge and SI Figs. S3 and S4 for the other bunch charges). For SASE radiation for all bunch charges we clearly observed a sharp peak corresponding to the spike shape staying on the pedestal of a broad peak corresponding to the spectrum bandwidth [see Fig. 3 ▸(*b*) and SI Figs. S3(*b*), S3(*d*) and S4(*b*)]. For the monochromatic beams we did not resolved individual spikes in the ACF and in the case of self-seeding a sharp peak corresponding to self-seeding radiation was staying on the broad pedestal. To determine the width of the peaks from the ACFs we performed Gaussian fits of the ACFs according to equation (6) (see Table 1 ▸).

From our ACF analysis we obtained the width of the spike in the case of SASE, monochromatic and self-seeding radiation to be about 0.4 ± 0.1 eV (FWHM) for all three bunch charges used in our experiment at the PAL-XFEL facility. We checked the obtained value by analysing the width of a few individual pulses and obtained the same value.

We further analysed the second-order correlation function in the spectral domain,

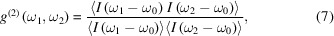

where ω_0_ is the central frequency.

**Figure 4:**
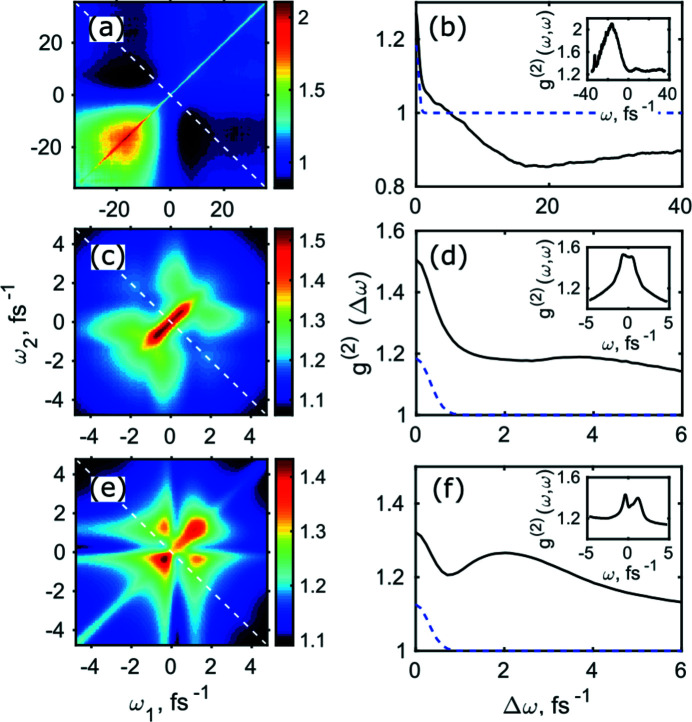
(*a*, *c*, *e*) Spectral second-order correlation function analysis at the 180 pC bunch charge. (*b*, *d*, *f*) Correlation functions *g* ^(2)^(Δω) (black lines) shown along the white dashed lines in (*a*, *c*, *e*). Blue dashed lines show the fit of the central peak in (*a*, *c*, *e*). (*a*, *b*) SASE radiation, (*c*, *d*) monochromatic radiation, (*e*, *f*) self-seeding regime of operation. (*b*, *d*, *f*) One-dimensional profiles along the white dashed lines in (*a*, *c*, *e*) shown by black lines. All results presented in this figure correspond to the 180 pC bunch charge.

The second-order correlation functions in the frequency domain for different operation conditions and 180 pC bunch charge are shown in Fig. 4 ▸ (see SI Figs. S9–S12 for the other bunch charges). As we clearly see from these results, the behaviour of the *g* ^(2)^(ω_1_, ω_2_) function is similar for the same modes of operation disregarding the bunch charge. For the SASE regime of operation, it has a narrow peak along the main diagonal with two maxima in the bottom left and top right positions [see Fig. 4 ▸(*a*) and SI Figs. S9(*a*) and S10(*a*)]. These maxima are an indication of the energy jitter as described by Gorobtsov *et al.* (2017 ▸).

The shape of the narrow peak may provide an estimate of the lower value of the average pulse duration. Unfortunately, in this particular experiment we did not measure pulse duration by any alternative methods such as, for example, using the cross-correlation method (Min *et al.*, 2019 ▸; Nam *et al.*, 2021 ▸; Ding *et al.*, 2012 ▸). We analysed the second-order correlation function *g* ^(2)^(ω_1_, ω_2_) in the frequency domain using the following expression (Vartanyants & Khubbutdinov, 2021 ▸; Khubbutdinov *et al.*, 2021 ▸),

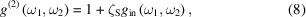

where ζ_S_ is the degree of spatial coherence and *g*_in_(ω_1_, ω_2_) is the correlation function in front of the on-line spectrometer. For the Gaussian Schell-model pulses, when the condition 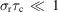 is satisfied, where σ_r_ is the r.m.s. value of the resolution function of the monochromator, we obtain for *g*_in_(ω_1_, ω_2_) in equation (8) (Vartanyants & Khubbutdinov, 2021 ▸),

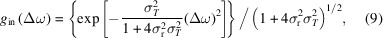

where σ_*T*_ is the r.m.s. of the average pulse duration, and the FWHM value can be obtained by expression *T* = 2(2ln2)^1/2^σ_*T*_ ≃ 2.355σ_*T*_. For the spectrometer resolution satisfying condition 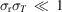 , equation (9) reduces to

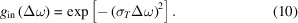

Taking into account the resolution of the spectrometer σ_r_ = 0.11 eV (Nam *et al.*, 2021 ▸), we determined from equation (9) the pulse duration for the SASE and monochromatic radiation to be from 6 fs to 9 fs depending on the operation conditions (see Table 1 ▸).

In the case of the self-seeding operation, the pulses were close to being transform limited with additional contribution of the SASE background. In addition, we observed a specific shape of the *g* ^(2)^(ω_1_, ω_2_) function in the form of a ‘leaf’ [see Fig. 4 ▸(*e*) and SI Fig. S10 for the bunch charge of 200 pC]. We leave the detailed analysis of the self-seeding mode until Section 4.

### 3.3. Spatial analysis

An average spatial intensity distribution, measured in the case of SASE radiation with the 180 pC bunch charge, is shown in Fig. 2 ▸(*c*). As one can see from this figure, there are some small artefacts and distortions that are present in this intensity distribution; these effects we attribute to imperfections of the KB mirrors. For the correlation analysis in the spatial domain we selected the region of interest that was about 1 mm × 1 mm (150 × 150 pixels), which is shown in Fig. 2 ▸(*c*) by the white dashed square.

The average spatial intensity distribution in the horizontal and vertical directions obtained for all pulses at the 180 pC bunch charge and SASE operation mode is shown in Fig. 2 ▸(*c*), which looks similar to other bunch charges. To estimate the FWHM size of the beam, we performed fitting by the Gaussian functions in the vertical and horizontal directions (see Table 2 ▸). In all cases the beam size was of the order of 0.7–1 mm (FWHM).

**Figure 5:**
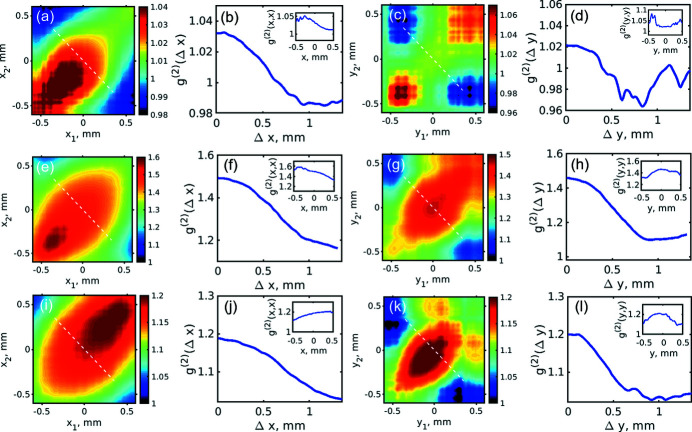
Intensity correlation functions *g* ^(2)^(*x*_1_,*x*_2_) (*a*, *e*, *i*) and *g* ^(2)^(*y*_1_,*y*_2_) (*c*, *g*, *k*) measured in the horizontal and vertical directions, respectively. Profiles of the *g* ^(2)^(Δ*x*) (*b*, *f*, *j*) and *g* ^(2)^(Δ*y*) (*d*, *h*, *l*) functions taken along the white dashed lines shown in panels (*a*, *e*, *i*) and (*c*, *g*, *k*), respectively. In the inset the corresponding autocorrelation functions *g* ^(2)^(*x*,*x*) and *g* ^(2)^(*y*,*y*) taken along the diagonal lines of *g* ^(2)^ functions are shown. (*a*–*d*) SASE radiation, (*e*–*h*) monochromatic radiation, (*i*–*l*) self-seeding regime of operation. All results presented in this figure correspond to the 180 pC bunch charge.

The second-order correlation analysis in this work was performed in the following way. We projected intensities for each pulse in the vertical and horizontal directions as 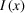 = 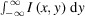 and 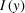 = 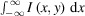 [see Fig. 2 ▸(*c*)]. Next, we correlated these projected intensities according to

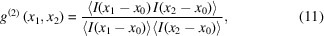

where *x*_0_ is the centre of mass of the projected intensity distribution and similar in the vertical direction. The results of intensity correlation analysis in the horizontal and vertical directions for all operation conditions with the 180 pC bunch charge are presented in Fig. 5 ▸ (see SI Figs. S13–S20 for the other bunch charges). The intensity correlation functions determined along the white dashed lines are also shown in Fig. 5 ▸. We observed that in the SASE operation regime we have two maxima along the diagonal in the bottom left and top right corners and a minimum in the middle. This is typical behaviour of the *g* ^(2)^ function in the case of positional jitter (Gorobtsov *et al.*, 2017 ▸). In the case of monochromatic radiation and the self-seeding regime of operation we observed that in most of the cases the maximum of distribution of the *g* ^(2)^ function is shifted from the centre. This effect may be due to the presence of two spatially separated beams in the intensity distribution (Gorobtsov *et al.*, 2017 ▸).

To obtain the values of the coherence length *L*_coh_, we extracted one-dimensional profiles along the white dashed lines shown in Fig. 5 ▸ and determined *L*_coh_ as variance values of these profiles (Khubbutdinov *et al.*, 2021 ▸),

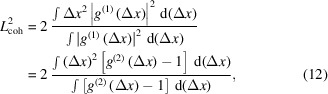

and similarly in the vertical direction. Equation (12) gives an exact result for the Gaussian distribution of the first-order correlation function 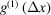 = 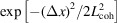 and integration from zero to infinity. The values of coherence length are summarized in Table 2 ▸. Here we should note that by deriving equation (12) we assume that equation (2) is valid. At the same time equation (2) was obtained under the conditions of chaotic radiation. This last assumption, as we know from our previous research (Singer *et al.*, 2013 ▸; Gorobtsov *et al.*, 2017 ▸, 2018*a* ▸; Khubbutdinov *et al.*, 2021 ▸), is valid for SASE radiation without and with the monochromator, but we should be careful by applying this approach to the self-seeding case. As we will discuss later, the contrast of the *g* ^(2)^ function is higher than that for the self-seeding mode of operation. However, we know that for fully coherent radiation the contrast should be equal to 1 (Gorobtsov *et al.*, 2018*a* ▸). So, finally, we applied the same equation (12) to estimate the coherence length also for the self-seeding mode of operation.

The obtained coherence length values (see Table 2 ▸) have to be compared with the r.m.s. values of the average beam size for each operation condition and evaluation direction. Such a comparison shows that in the case of SASE radiation the coherence length is of the order of the r.m.s. values of the average beam size, and in the case of the monochromatic and self-seeding operation the coherence length exceeds the r.m.s. values of the average beam size. From this we can deduce that the degree of coherence is already high in the SASE regime of operation and is substantially higher in the case of the monochromatic and self-seeding operation conditions.

Next, the degree of spatial coherence ζ_S_ in each transverse direction was determined. The degree of spatial coherence for a chaotic source can be determined according to the following equation (Gorobtsov *et al.*, 2017 ▸),

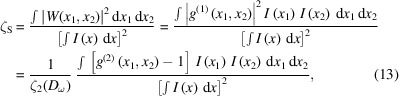

where *W*(*x*_1_, *x*_2_) is the cross-spectral density function. The degree of spatial coherence took values in the range from 50% to 81% depending on the operation regime and electron bunch charge (see Table 2 ▸). In the SASE operating regime, the degree of spatial coherence was in most cases in the range from 50% to 70%. For the monochromatic regime of operation, the degree of spatial coherence was of the order of 80% and did not depend strongly on the bunch charge. For the self-seeding regime, it was also of the order of 80% as in the monochromatic case and was slightly growing with the bunch charge. The degree of spatial coherence for the Gaussian Schell-model may be determined also from the following equation (Vartanyants & Singer, 2010 ▸, 2020 ▸),

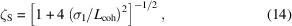

where σ_I_ is the r.m.s. value of the averaged intensity distribution and *L*_coh_ is the coherence length.

The contrast values 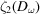 [see equation (2)] for all operating conditions were deduced directly from the *g* ^(2)^ function as 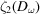 = 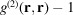 at **r** = 0 in the horizontal and vertical directions (see Table 2 ▸). These values of the contrast are directly related to the degree of coherence in the spectral domain (Vartanyants & Khubbutdinov, 2021 ▸). In the SASE operation regime, the contrast values were from 1% to 6% depending on the bunch charge and evaluation direction. These small values concord well with an estimate for the contrast given by the following relation, 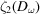 ≃ 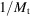 , where *M*_t_ is the number of longitudinal modes. Our estimate of the number of longitudinal modes *M*_t_ for the SASE radiation shows that it is quite high and is about 40 modes for bunch charges of 120 pC and 200 pC (see SI Table S3). At the same time, we found quite high numbers of modes of ∼100 for the 180 pC bunch charge, that provides the low value of the contrast. As soon as the number of modes is substantially decreased for the monochromatic operation (three to five modes) (see SI Table S3), we expect an increase in the values of contrast for these operation conditions of the PAL-XFEL. As follows from Table 2 ▸, the contrast values for the monochromatic radiation are in the range 25–40%, which match well with the estimated number of modes. Interestingly, for the self-seeding mode of operation the contrast values are in the range 5–22% depending on the bunch charge. They are obviously lower than in the monochromatic case but are still not zero. We will discuss these observations in the next section.

## 4. Discussion

### 4.1. Degree of coherence

As a result of our HBT analysis, we obtained a high degree of coherence in the range 50–70% for the SASE radiation in the spatial domain. The degree of coherence in the monochromatic and self-seeding operation regimes was even higher and was in the range 75–85%. It is interesting to note that the degree of coherence in the monochromatic and self-seeding operation regimes were quite similar.

### 4.2. Self-seeding operation mode

Now we turn to our basic question that was formulated in the beginning of this work: whether radiation in the self-seeding mode is fully coherent or rather has chaotic nature. To address this question, we turn our attention to the results obtained for the contrast of the spatial analysis. From our analysis we observed quite low contrast in the case of SASE radiation. This is expected, due to the large amount of temporal modes *M*_t_ present in each XFEL pulse. As the number of modes is reduced by applying a monochromator, we obtain a significant increase in the contrast values. However, when we turn to the self-seeding mode of operation, the results are quite different from the previous one. At the 120 pC bunch charge, we observe low values of contrast of about 5%, indicating that at this bunch charge radiation is rather coherent [compare with the results obtained at the externally seeded FEL FERMI (Gorobtsov *et al.*, 2018*a* ▸)]. At the same time, at the 180 pC and 200 pC bunch charges we observed that the contrast values are about 20%, which is lower than in the monochromatic case, but sufficiently larger than in the SASE case. From these results we can conclude that, in the case of self-seeding, radiation is in a mixed state: it is not fully coherent, but it is also not fully chaotic. The balance between these competing terms may be different depending on the specific tuning of the PAL-XFEL machine for this particular experiment.

### 4.3. Pulse duration

In addition, from our HBT analysis in the frequency domain we obtained comparably short pulse durations in the range 6–9 fs, which were substantially shorter than reported earlier (Kang *et al.*, 2019 ▸; Yun *et al.*, 2019 ▸). There may be several reasons for this. For example, our results do not take into account broadening of the spectrum due to frequency chirp effects or the electron bunch compression factor (Krinsky & Li, 2006 ▸). If the electron beam is chirped, this will bring in turn a broadening of the spectrum of the generated radiation. As was shown in our previous work (Khubbutdinov *et al.*, 2021 ▸), frequency chirp effects could mean a substantial lower value of the pulse durations from the HBT analysis.

### 4.4. Simulations

**Figure 6:**
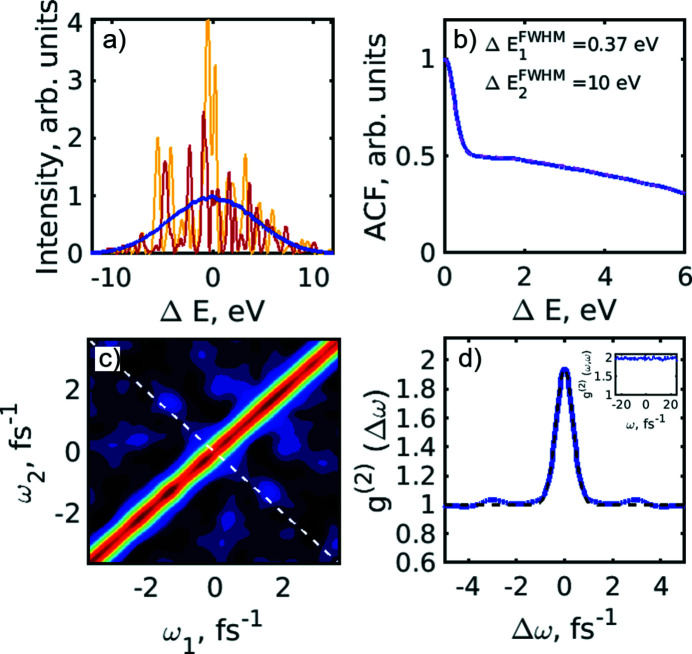
Initial simulation in the spectral domain. (*a*) Typical single-shot simulated spectra and an averaged spectrum (blue line). (*b*) Autocorrelation function of the individual spectral lines averaged over 5 × 10^3^ pulses (blue solid line) and the fit with the two Gaussian functions (magenta dashed line). (*c*) Intensity correlation function *g* ^(2)^(ω_1_,ω_2_) of the simulated spectra. (*d*) Intensity correlation function *g* ^(2)^(Δω) (blue line) taken along the white dashed line in (*c*) and its fit (black dashed line) with equation (9). In the inset the profile of the *g* ^(2)^(ω,ω) function along the diagonal in (*c*) is shown.

In order to better understand some statistical features of the radiation produced by the PAL-XFEL facility and revealed by our HBT analysis, we performed some additional simulations, where we used an approach based on work by Pfeifer *et al.* (2010 ▸) [see also Khubbutdinov *et al.* (2021 ▸)]. The stochastic XFEL radiation in the time–frequency domain with 5 × 10^3^ pulses was generated by this method for each particular simulation case. For the initial simulation, the average spectrum was considered to be Gaussian and centred at the frequency ω_0_, corresponding to a resonant energy of *E*_0_ = 10 keV. The spectral width was considered to be Δ*E*_FWHM_ = 10 eV as in SASE radiation in our experiment. The profile of the pulse in the time domain was considered to be Gaussian with the pulse duration *T*_FWHM_ = 5 fs. Results of these simulations are shown in Fig. 6 ▸. Typical single-shot simulated spectra and an averaged spectrum, as well as an autocorrelation function averaged over the individual spectral lines, are shown in Figs. 6 ▸(*a*) and 6(*b*). The ACF analysis showed the FWHM size of the average spectrum to be 10 eV (as initially considered in the simulation), and the FWHM of the single spectral spike was 0.4 eV (similar to the SASE radiation case in our experiment). Analysing the variation of the integrated spectral intensity distribution, we determined the number of modes present in the simulated SASE spectrum to be about *M* = 28. The second-order intensity correlation function of the simulated spectra *g* ^(2)^(ω_1_,ω_2_) is shown in Fig. 6 ▸(*c*). The cut of this distribution along the diagonal line, shown by the white dashed line in Fig. 6 ▸(*c*), is presented in Fig. 6 ▸(*d*). This distribution, *g* ^(2)^(*Δω*), was fitted according to equations (8) and (10) and provided the initial pulse duration of 5 fs.

**Figure 7:**
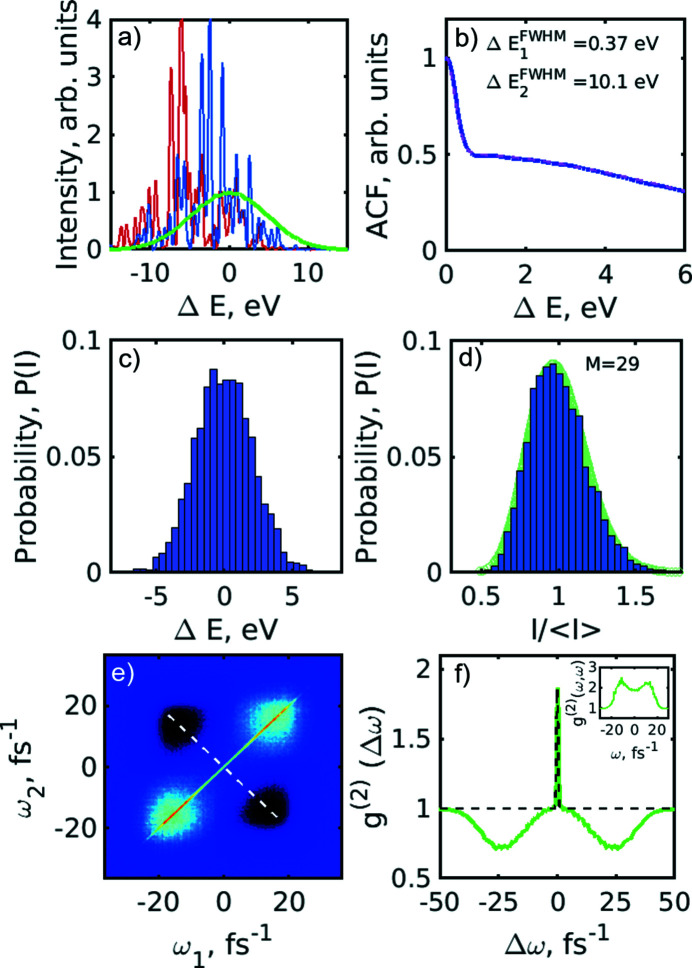
Spectral analysis simulation with a resonant energy jitter of 5 eV (FWHM). (*a*) Typical single-shot simulated spectra and an averaged spectrum (green line). (*b*) Autocorrelation function of the individual spectral lines averaged over 5 × 10^3^ pulses (blue solid line) and the fit with the two Gaussian functions (magenta dashed line). (*c*) Histogram of the resonant energy distribution. (*d*) Histogram of the spectral pulse intensity distribution (blue). The green background corresponds to the gamma probability distribution function with number of modes *M* = 29. (*e*) Second-order intensity correlation function *g* ^(2)^(ω_1_,ω_2_) of the simulated spectra. (*f*) Second-order intensity correlation function *g* ^(2)^(Δω) taken along the white dashed line in (*c*) and its fit (black dashed line) with equation (9). In the inset the profile of the *g* ^(2)^(ω,ω) function along the diagonal in (*e*) is shown.

Since the FEL is a complicated machine, many instabilities may arise during the electron bunch acceleration and radiation amplification process. Results of such instabilities can manifest themselves, for example, in the resonant energy jitter. To study this energy jitter on the *g* ^(2)^ correlation functions, the resonant energy of 10 keV was allowed to have variations of 5 eV (FWHM) photon energy according to the Gaussian distribution (see Fig. 7 ▸). As a result of these simulations we observed that the *g* ^(2)^(*Δω*) correlation function along the anti-diagonal line went below 1 and at the same time the *g* ^(2)^(*ω,ω*) correlation function along the diagonal line showed an increase in intensity [see Figs. 7 ▸(*e*) and 7(*f*)]. Both these effects were similar to those observed in our experiment [compare with Figs. 4 ▸(*a*) and 4(*b*)]. The above-mentioned features indicate the presence of the energy jitter effects in our experiment. In addition, we observed that the estimated pulse duration has changed by 0.3 fs or by 6% of the initial pulse duration (*T*_FWHM_ = 5 fs) assumed in the simulations.

**Figure 8:**
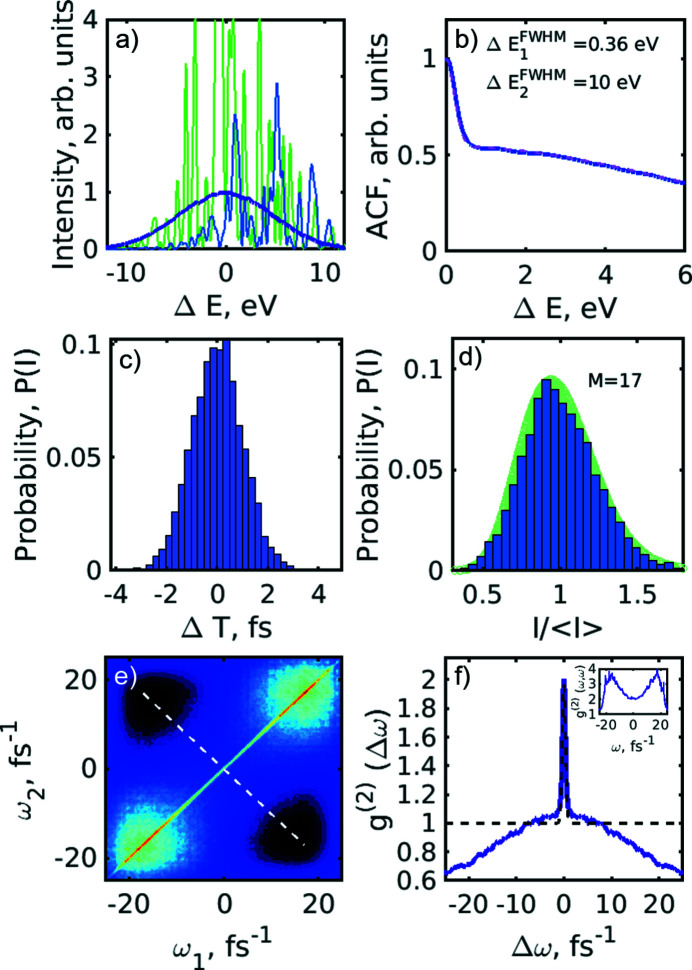
Spectral analysis simulation with an energy jitter of 5 eV (FWHM) and additional pulse duration jitter of 1 fs (r.m.s.). (*a*) Typical single-shot simulated spectra and an averaged spectrum (blue line). (*b*) Autocorrelation function of individual spectral lines averaged over 5 × 10^3^ pulses (blue line) and the fit with the two Gaussian functions (magenta dashed line). (*c*) Histogram of the pulse duration distribution. (*d*) Histogram of the spectral pulse intensity distribution (blue). The green background corresponds to the gamma probability distribution function with number of modes *M* = 17. (*e*) Second-order intensity correlation function *g* ^(2)^(ω_1_,ω_2_) of the simulated spectra. (*f*) Second-order intensity correlation function *g* ^(2)^(Δω) taken along the white dashed line in (*c*) and its fit (black dashed line) with equation (9). Additional features around Δω = 0 are easily seen in this panel. In the inset the profile of the *g* ^(2)^(ω,ω) function along the diagonal in (*e*) is shown.

Along with the energy jitter, the pulse duration jitter from pulse to pulse might also affect the observed *g* ^(2)^(ω_1_,ω_2_) correlation function. To study this effect, we simulated pulses with 1 fs (r.m.s.) variations from pulse to pulse following a Gaussian distribution. As a result of these simulations, a small ‘bump’ in the distribution of the *g* ^(2)^(*Δω*) correlation function was observed [see Fig. 8 ▸(*f*)], which was similar to our experimental results [see Fig. 4 ▸(*b*)]. The presence of such a broadening in the correlation functions obtained from our experimental data may indicate a possible pulse duration jitter at the PAL-XFEL facility. In addition to the broadening of the *g* ^(2)^(*Δω*) correlation function, we observed that the pulse duration has changed by 6% of the initial pulse duration assumed in the simulations.

**Figure 9:**
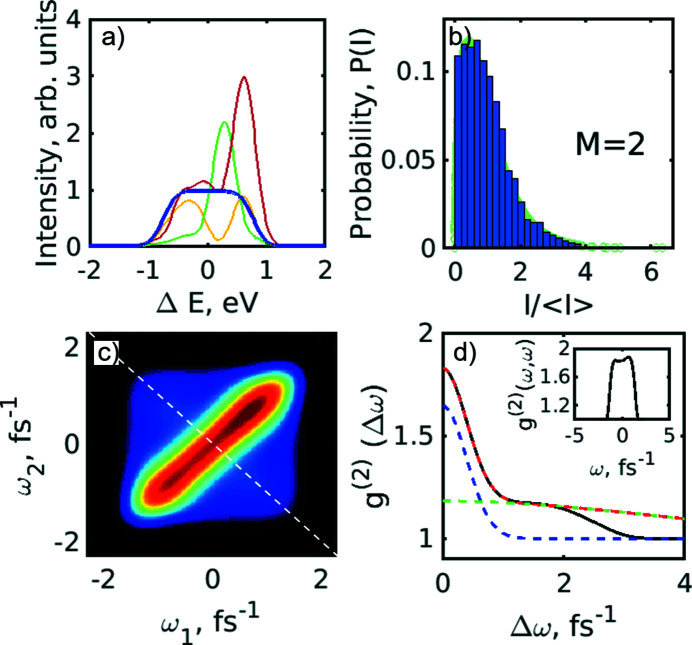
Spectral analysis simulation with the monochromator installed in the beamline. (*a*) Simulated profiles of single-pulse intensities and an averaged spectrum (blue line) with a bandwidth of 1.9 eV. (*b*) Histogram of intensities of single pulses obeying the gamma function distribution (green background) with number of modes *M* = 2. (*c*) Second-order intensity correlation function *g* ^(2)^(ω_1_,ω_2_) of the simulated spectra with the monochromator. (*d*) Second-order intensity correlation function *g* ^(2)^(Δω) (black curve) taken along the white dashed line in (*c*) and its fit (red dashed line) with equation (9). In the inset the profile of the *g* ^(2)^(ω,ω) function along the diagonal in (*c*) is shown.

We also simulated results of monochromatic radiation on the *g* ^(2)^ function. For this, for generated pulses we applied a bandwidth of Δ*E* = 1.9 eV in the frequency domain (see Fig. 9 ▸). In the distribution of modes we obtained only two modes that considerably contribute to the result. For the *g* ^(2)^(ω_1_,ω_2_) correlation function we obtained the result shown in Fig. 9 ▸(*c*) that is similar to our experimental result for the monochromatic case [see Fig. 4 ▸(*c*)].

**Figure 10:**
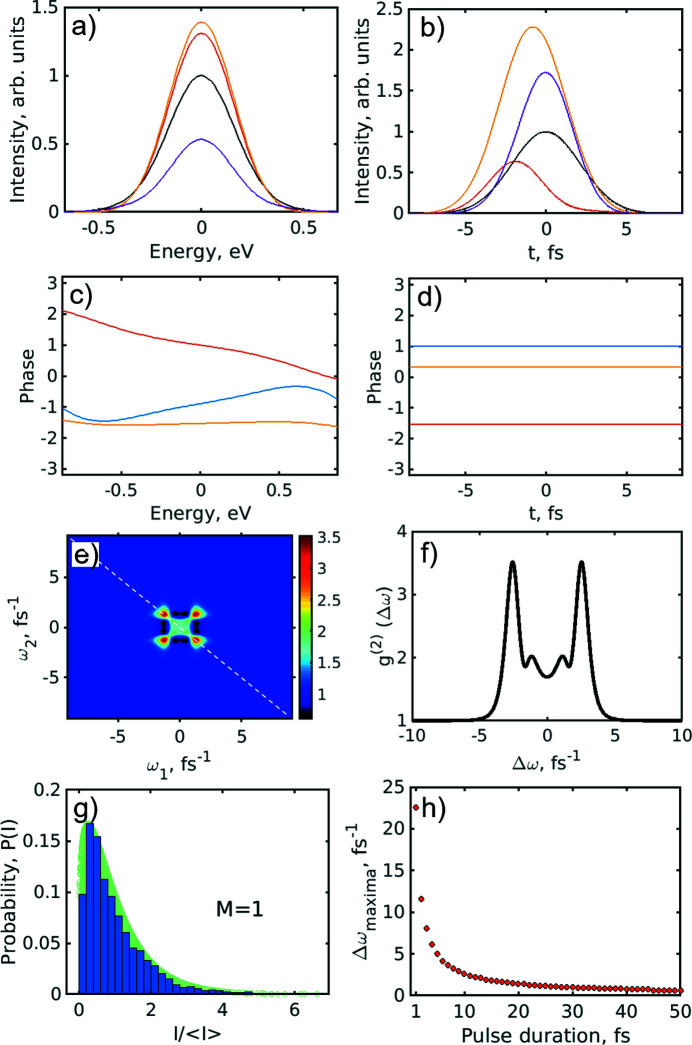
Spectral simulations for the seeded beam. (*a*–*d*) Example of generated single pulses in (*a*) the energy domain with bandwidth 0.4 eV and (*b*) the time domain with pulse duration *T* = 5 fs (FWHM). (*c*, *d*) The corresponding phases for these pulses in the energy domain (*c*) and in the time domain (*d*). The phases in the time domain were put to a constant value corresponding to phase at *T* = 0. (*e*) Second-order intensity correlation function *g* ^(2)^(ω_1_,ω_2_) of the simulated spectra of the seeded beam. (*f*) Second-order intensity correlation function *g* ^(2)^(Δω) (black curve) taken along the white dashed line in (*e*). (*g*) Histogram of intensities of single pulses obeying the gamma function distribution (green background) with number of modes *M* = 1. (*h*) Distance between two maxima Δω_maxima_ in the *g* ^(2)^(Δω) correlation function in (*f*) as a function of pulse duration.

Next, we turned to the simulation of the self-seeded pulses. We used the same approach and fixed the pulse duration to be about *T* = 5 fs and, at the same time, reduced the bandwidth of the generated pulses in the frequency domain [*ΔE* = 0.4 eV (FWHM)] until we obtained a single mode distribution [see Fig. 10 ▸(*g*)]. It is interesting to note that each pulse in this simulation had a varying phase both in the energy and time domains that was random from pulse to pulse [see Fig. 10 ▸(*c*)]. Then the pulses were modified in the time domain by putting a constant value to the phases of each pulse and allowing these phases to change randomly from pulse to pulse [see Fig. 10 ▸(*d*)]. To our surprise in this case we obtained the shape of the *g* ^(2)^(ω_1_,ω_2_) correlation function in the form of a leaf [see Fig. 10 ▸(*e*)], similar to our results for the self-seeding operation mode [see Fig. 4 ▸(*e*)]. We also noticed that the distance between two maxima along the anti-diagonal line depends on the pulse duration. To analyse this in detail we plotted this distance in frequency Δω as a function of pulse duration and obtained the curve shown in Fig. 10 ▸(*h*). From that curve we identified that for the distance between two maxima determined from our experiment (Δω = 3.5 fs^−1^) we obtain a pulse duration for the self-seeding operation mode of about 7 fs.

## 5. Summary

In summary, statistical analysis was performed to characterize hard X-ray radiation at PAL-XFEL by the HBT interferometer technique. In particular, information on average energy distribution, coherence time and pulse duration could be obtained by spectral analysis, and information on beam size, coherence length and degree of coherence could be obtained by spatial intensity analysis under various conditions (SASE, monochromatic and self-seeded radiation at 120 pC, 180 pC, and 200 pC bunch charges).

The results of this experiment not only allow us to understand the present performance of PAL-XFEL but will be an important factor for facility upgrades in the future.

## Supplementary Material

Sections S1 to S6; Figures S1 to S20; Tables S1 to S3. DOI: 10.1107/S1600577522008773/yi5125sup1.pdf

## Figures and Tables

**Table 1:** Results of the analysis in the spectral domain for the 120, 180 and 200 pC bunch charges used in the experiment The XFEL spectrum bandwidth was obtained directly from the averaged spectrum of all pulses in each operation condition. Analysis of the autocorrelation function (ACF) was performed using equation (6) that provided the intrinsic XFEL spectrum bandwidth as well as the spike bandwidth. Coherence times were estimated from equation (5) assuming that the average spectrum is fitted by two Gaussian functions. Pulse duration was obtained by fitting the central part of the second-order correlation function *g*_in_(*Δω*) in equation (9). The values of different parameters measured in the linear mode of operation, indicated as (L), are also provided.

	120 pC			180 pC			200 pC		
Operation mode	SASE radiation	Mono-chromatic radiation	Self-seeding radiation	SASE radiation	Mono-chromatic radiation	Self-seeding radiation	SASE radiation	Mono-chromatic radiation	Self-seeding radiation
XFEL spectrum bandwidth (FWHM) (eV)	11.9 / 13.3 (L)	1.2 / 1.2 (L)	0.4	11.5	1.1	0.4	27.8	1.1	0.4 / 0.5 (L)
XFEL spectrum bandwidth from ACF (FWHM) (eV)	12.41 ± 0.3 / 12.0 ± 0.1 (L)	1.37 ± 0.1 / 1.37 ± 0.1 (L)	3.09 ± 0.1	12.6 ± 0.1	1.3 ± 0.1	2.9 ± 0.1	24.0 ± 0.2	1.3 ± 0.2	3.6 ± 0.1 / 4.1 ± 0.1 (L)
Spike bandwidth from ACF (FWHM) (eV)	0.4 ± 0.1 / 0.4 ± 0.1 (L)	0.4 ± 0.1 / 0.4 ± 0.1 (L)	0.4 ± 0.1	0.4 ± 0.1	0.4 ± 0.1	0.4 ± 0.1	0.4 ± 0.1	0.4 ± 0.1	0.4 ± 0.1 / 0.54 ± 0.1 (L)
Coherence time (r.m.s.) (fs)	0.17 ± 0.01 / 0.17 ± 0.01 (L)	2.55 ± 0.03 / 2.56 ± 0.03 (L)	4.64 ± 0.03	0.17 ± 0.01	2.48 ± 0.28	4.19 ± 0.10	0.11 ± 0.01	2.25 ± 0.06	3.68 ± 0.15 / 3.44 ± 0.04 (L)
Pulse duration *T* (fs)	6.0 ± 0.2 / 6.1 ± 0.2 (L)	7.2 ± 0.2 / 6.0 ± 0.2 (L)	–	7.0 ± 0.2	8.8 ± 0.2	–	6.4 ± 0.2	7.2 ± 0.2	–

**Table 2:** Results of the analysis in the spatial domain for the 120, 180 and 200 pC bunch charges used in the experiment The beam size (FWHM) was determined by the direct evaluation of an averaged intensity distribution. The coherence length *L*_coh_ was obtained from equation (12) in which integration was performed over the region where *g* ^(2)^(Δ**r**) ≥ 1. The degree of coherence was determined by equation (13) in which integration was performed over the region where *g* ^(2)^(Δ**r**) ≥ 1. The contrast value was defined as 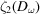 = 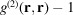 at **r** = 0 in the horizontal and vertical directions, respectively. The values of different parameters measured in the linear mode of operation are also provided.

	120 pC			180 pC			200 pC		
Operation mode	SASE radiation	Mono-chromatic radiation	Self-seeding radiation	SASE radiation	Mono-chromatic radiation	Self-seeding radiation	SASE radiation	Mono-chromatic radiation	Self-seeding radiation
Horizontal direction, *x*									
Average beam size (FWHM) (mm)	0.85 ± 0.02 / 1.02 ± 0.01 (L)	0.77 ± 0.01 / 0.95 ± 0.01 (L)	0.70 ± 0.01	0.74 ± 0.00	0.75 ± 0.01	0.72 ± 0.01	0.83 ± 0.01	0.79 ± 0.02	0.73 ± 0.00 / 0.84 ± 0.00 (L)
Coherence length (r.m.s.) (mm)	0.18 ± 0.02 / 0.22 ± 0.02 (L)	0.46 ± 0.04 / 0.67 ± 0.06 (L)	0.51 ± 0.06	0.41 ± 0.04	0.9 ± 0.08	0.71 ± 0.06	0.5 ± 0.04	0.61 ± 0.05	0.7 ± 0.06 / 0.8 ± 0.07 (L)
Degree of coherence, ζ (%)	51.2 ± 0.90 / 31.6 ± 0.1 (L)	76.0 ± 3.9 / 80.2 ± 1.6 (L)	79.0 ± 2.9	60.6 ± 1.2	80.6 ± 2.7	80.4 ± 1.3	68.5 ± 1.6	78.6 ± 6.0	84.2 ± 0.5 / 91.9 ± 0.9 (L)
Contrast	0.06 ± 0.01 / 0.02 ± 0.01 (L)	0.29 ± 0.03 / 0.21 ± 0.03 (L)	0.05 ± 0.01	0.02 ± 0.00	0.42 ± 0.08	0.19 ± 0.01	0.04 ± 0.00	0.35 ± 0.07	0.22 ± 0.02 / 0.13 ± 0.02 (L)
Vertical direction, y									
Average beam size (FWHM) (mm)	0.52 ± 0.00 / 0.61 ± 0.00 (L)	0.56 ± 0.00 / 0.67 ± 0.01 (L)	0.45 ± 0.00	0.48 ± 0.00	0.52 ± 0.01	0.46 ± 0.01	0.52 ± 0.00	0.54 ± 0.01	0.45 ± 0.00 / 0.50 ± 0.00 (L)
Coherence length (r.m.s.) (mm)	0.49 ± 0.04 / 0.33 ± 0.03 (L)	0.46 ± 0.04 / 0.45 ± 0.04 (L)	0.25 ± 0.02	0.28 ± 0.02	0.68 ± 0.06	0.59 ± 0.05	0.43 ± 0.04	0.61 ± 0.05	0.6 ± 0.05 / 0.6 ± 0.05 (L)
Degree of coherence, ζ (%)	70.8 ± 2.2 / 84.3 ± 3.5 (L)	84.0 ± 0.8 / 90.4 ± 0.9 (L)	68.3 ± 2.0	72.9 ± 0.9	83.3 ± 0.8	76.8 ± 1.7	68.2 ± 0.8	81.6 ± 1.3	80.5 ± 0.6 / 88.0 ± 1.0 (L)
Contrast	0.04 ± 0.01 / 0.01 ± 0.00 (L)	0.26 ± 0.02 / 0.18 ± 0.02 (L)	0.06 ± 0.01	0.01 ± 0.00	0.40 ± 0.07	0.20 ± 0.01	0.04 ± 0.00	0.34 ± 0.06	0.23 ± 0.02 / 0.14 ± 0.02 (L)

## Footnotes

1 It was shown theoretically that statistical properties of radiation may deviate from Gaussian statistics for radiation in the saturation region (Saldin *et al.*, 2010 ▸).

2 For a description of the Andor detector, see https://pdf.medicalexpo.com/pdf/andor-technology-plc/zyla-55-hf/90363-177120.html.

3 For a description of the Hamamatsu detector, see https://www.hamamatsu.com/eu/en/product/cameras/x-ray-cmos-cameras/C12849-111U.html.
