# Supplementary material for: Statistical analysis of hard X-ray radiation at the PAL-XFEL facility performed by Hanbury Brown and Twiss interferometry
Source: J Synchrotron Radiat. 2022 Oct 7;29(Pt 6):1465–79. doi: 10.1107/S1600577522008773 (PMC9641567; doi:10.1107/S1600577522008773)
Supplement: Supplementary file 1 [file s-29-01465-sup1.pdf]

JOURNAL OF SYNCHROTRON RADIATION

Volume 29 (2022)

Supporting information for article:

Statistical analysis of hard X-ray radiation at PAL-XFEL facility performed by Hanbury Brown and Twiss interferometry

Young Yong Kim, Ruslan Khubbutdinov, Jerome Carnis, Sangsoo Kim, Daewoong Nam, Inhyuk Nam, Gyujin Kim, Chi Hyun Shim, Haeryong Yang, Myunghoon Cho, Chang-Ki Min, Changbum Kim, Heung-Sik Kang and Ivan Vartanyants

S1. Data processing

In this experiment, we simultaneously measured intensity information in the spectral and spatial domains. The number of pulses analysed in this work for each bunch charge and operation condition of the PAL XFEL are listed in Table S1. In order to extract accurate information of the data, dark images of about 1000 shots were obtained in each condition, the average value was calculated, and the dark image was subtracted from each data in spectral and spatial domains. After processing of these data, a projection along the vertical direction was performed in 2D spectral detector. One example is shown in Fig. S1 (a,b). In each condition, the average profile from all pulses of the one-dimensional spectra was obtained, the zero value of ∆E was set through the fitting of Gaussian distribution, and the value of full width of half maximum (FWHM) for energy distribution was obtained (see Fig. S1 (c)).

For each pulse, a spatial image was also projected vertically and horizontally to obtain the one-dimensional profile. After that, the average value for all pulses was calculated, the zero position was set through the fitting of the Gaussian distribution in each direction, and the FWHM for the beam size was calculated (see Fig. S1 (e,f)).

Table S1 Number of pulses that was analysed in this work.

Bunch charge	120 pC			180 pC			200 pC		
Operation mode	SASE radiation	Mono-chromatic radiation	Self-seeding radiation	SASE radiation	Mono-chromatic radiation	Self-seeding radiation	SASE radiation	Mono-chromatic radiation	Self-seeding radiation
Number of pulses	9,833 /16,876 (L)	11,403 /19,961 (L)	19,938	11,866	9,513	19,869	7,940	8,405	19,781 /19,936 (L)

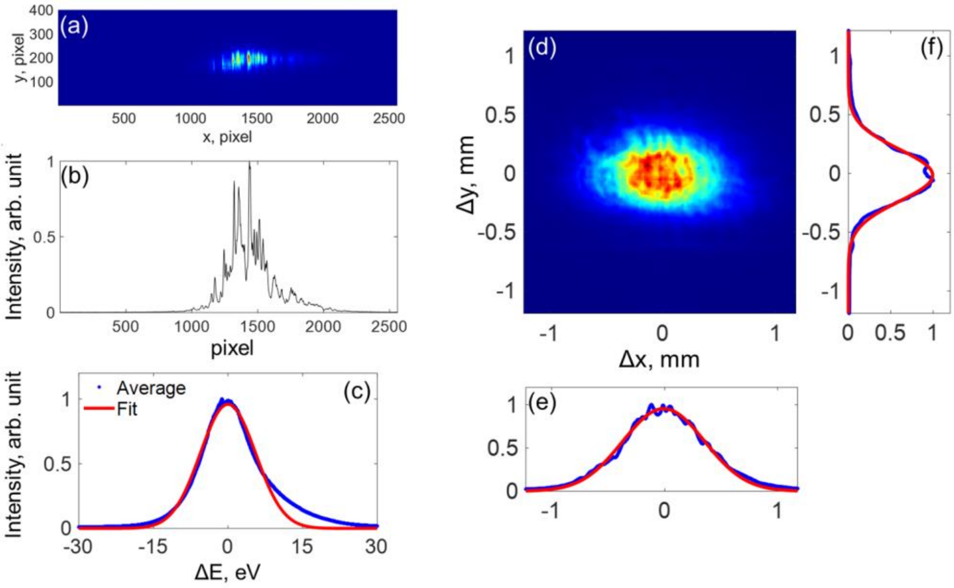

Figure S1 Results of the spectrum and intensity distributions in spatial domain for SASE radiation at 120 pC bunch charge. From all shown images an average dark background from 1,000 pulses was subtracted. (a) Image of a single spectrum, (b) projection of this spectrum along the vertical direction, (c) average spectral intensity (blue line) and the fit with the Gaussian function (red line). (d) Average intensity at Hamamatzu detector. (e,f) Projection of this intensity in the vertical (e) and horizontal (f) directions. Blue lines are the average intensities for each projection and red lines are the fit with the Gaussian function.

S2. Monochromator drift corrections

During the measurements for the case of SASE with the monochromator, we observed the vertical position drifts of the monochromator (see Fig. S2). So, we set the sections where the drift was happening rapidly, calculated the linear regression for each section, and then corrected it (see Fig. S2 (b,c)). This correction was performed for all monochromatic radiation modes.

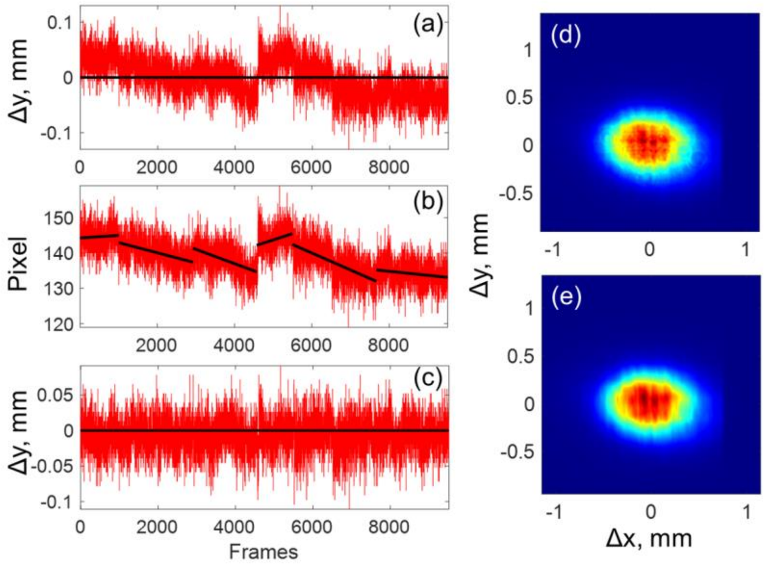

Figure S2 Spatial corrections for the monochromator mode of operation at the 180 pC bunch case. (a) Centre of mass position along the vertical direction at each frame. (b) linear regression at each selected region. (c) Centre of mass position after correction. Average spatial intensity before correction (d) and after correction (e).

S3. Spectral profile analysis

The 200 pulses of the one-dimensional single spectral profiles, an average profile, and the autocorrelation function (ACF) for all operating conditions at 120 pC and 200 pC are shown in Figs. S3-S4. The analysis of the ACF determined the bandwidth of the spectrum related to the average FEL bandwidth as well as a single spike width (Khubbutdinov *et al.*, 2021). The averaged ACF was obtained by calculating the autocorrelation functions for each spectrum individually and then averaging it over all pulses, for certain conditions of operation of the PAL XFEL. To extract the FWHM values from the ACF's for all operation conditions, we performed the Gaussian function fitting. The average FWHM width of the peaks in spectra ΔE is related to the width of the peaks determined from the autocorrelation function ΔE_{ACF} as $\Delta E = \Delta E_{ACF}/\sqrt{2}$.

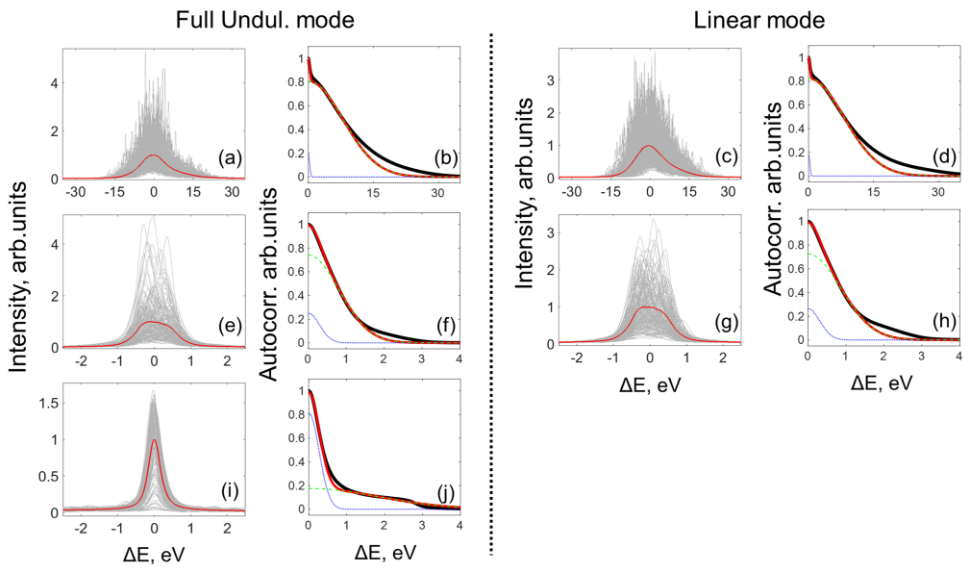

Figure S3 (a, e, i) Spectral distribution of 200 pulses (grey lines) and an average spectrum for all pulses (red lines). (b, f, g) Average autocorrelation functions of all spectral lines (black lines), and its fit (red lines) by two Gaussian functions, broad (green dashed lines) and narrow (blue dotted lines). (a,b) SASE radiation, (e,f) SASE monochromatic radiation, (i,j) self-seeding regime of operation. (c,d) Same in SASE linear mode of operation, (g,h) Same in SASE linear mode of operation with the monochromator. All results presented in this figure correspond to the 120 pC bunch charge.

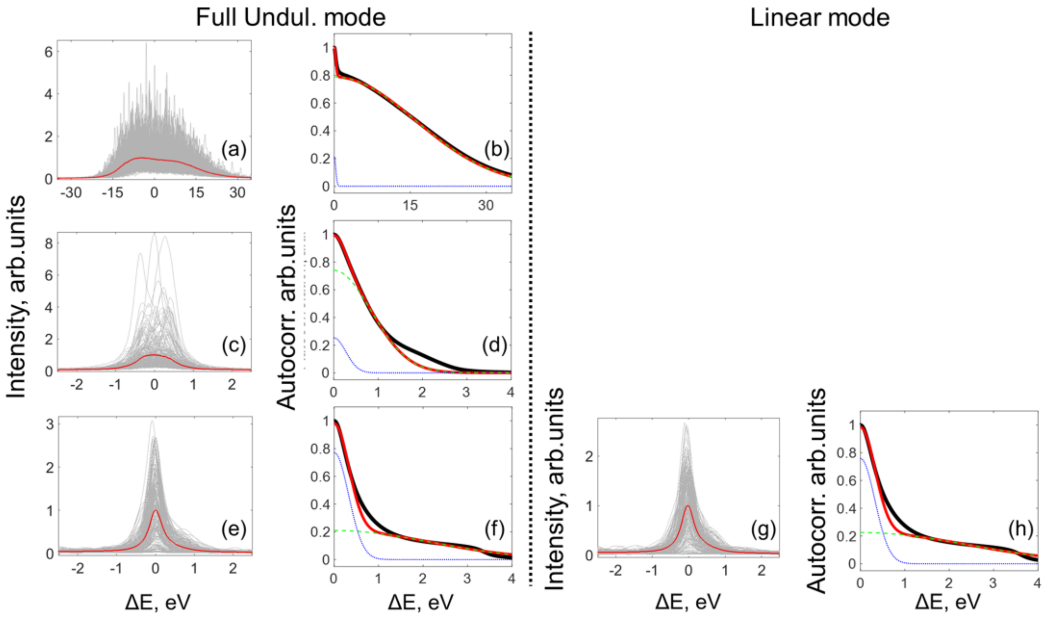

Figure S4 (a, c, e) Spectral distribution of 200 pulses (grey lines) and an average spectrum for all pulses (red lines). (b, d, f) Average autocorrelation functions of all spectral lines (black lines), and its fit (red lines) by two Gaussian functions, broad (green dashed lines) and narrow (blue dotted lines). (a,b) SASE radiation, (e,f) SASE monochromatic radiation, (i,j) self-seeding regime of operation.

(g,h) Same in self-seeding linear mode of operation. All results presented in this figure correspond to the 200 pC bunch charge.

S4. An average spectral profile fit

As it was described in the main text to determine coherence time one needs to obtain an averaged spectral profile. We noticed that in the case of PAL XFEL an averaged spectral profile is well fitted by two Gaussian functions as

$$S(\omega) = A_1 exp\left[-\frac{(\omega-\omega_1^0)^2}{2\sigma_1^2}\right] + A_2 exp\left[-\frac{(\omega-\omega_2^0)^2}{2\sigma_2^2}\right], \quad \text{(S1)}$$

where A_1 and A_2 are scaling coefficients, $\boldsymbol{\omega_1^0}$ and $\boldsymbol{\omega_2^0}$ are the centres of each Gaussian line and σ_1 and σ_2 are their rms values. Results of such fitting are summarized in Table S2 and shown in Fig. S5. Substituting this spectral profile in Eqs. (3,4) of the main text one can obtain for the coherence time an expression given in Eq. (5) of the main text (Khubbutdinov *et al.*, 2021).

Table S2 Parameters of the two Gaussian functions obtained from the fit of the averaged spectrum in different operation conditions (see Eq. (S1)).

Bunch charge	120 pC			180 pC			200 pC		
Operation mode	SASE radiation	Mono-chromatic radiation	Self-seeding radiation	SASE radiation	Mono-chromatic radiation	Self-seeding radiation	SASE radiation	Mono-chromatic radiation	Self-seeding radiation
A_1	0.657 /0.590(L)	0.761 /0.741(L)	0.744	0.701	0.533	0.692	0.469	0.749	0.646 /0.639(L)
ω_1^0, eV	-0.941 /-1.745(L)	-0.413 /-0.435(L)	0.004	-0.846	-0.548	-0.016	-11.497	-0.240	0.005 /-0.045(L)
σ_1, eV	6.081 /6.428(L)	0.465 /0.472(L)	0.216	5.925	0.428	0.215	7.219	0.554	0.212 /0.219(L)
A_2	0.354 /0.440(L)	0.735 /0.723(L)	0.234	0.316	0.879	0.301	0.851	0.442	0.327 /0.336(L)
ω_2^0, eV	4.147 /4.476(L)	0.506 /0.474(L)	0.050	5.000	0.307	0.159	6.538	0.545	0.110/ 0.074 (L)
σ_2, eV	14.149 /12.920 (L)	0.525 /0.520(L)	0.652	14.993	0.550	0.633	16.170	0.526	0.711 /0.754(L)

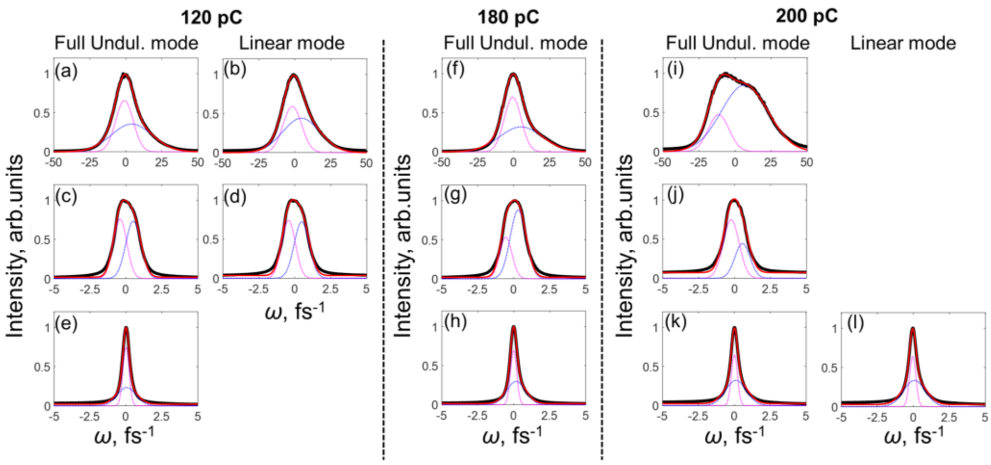

Figure S5 Results of fitting (red line) of an averaged spectrum (black line) by two Gaussian functions (magenta and blue lines) for different operation conditions. (a, f, i) SASE mode, (b) SASE linear mode, (c, g, j) SASE monochromatic conditions, (d) SASE monochromatic linear mode, (e, h, k) self-seeding mode, and (l) self-seeding linear operation mode. Measurements were performed at the 120 pC (a – e), 180 pC (f – h), and 200 pC (i – l) bunch charges, respectively.

S5. Intensity histograms and calculation of the number of modes

To analyse the statistical behaviour of FEL radiation we looked on the histograms of the pulse intensity distributions. These histograms were evaluated for all operation conditions from spectral and spatial measurements of PAL XFEL source and are presented in Figs. S6- S8. The histograms were compared with the Gamma probability distribution function which describes statistical behaviour of the FEL SASE radiation in the linear regime of operation (Saldin *et al.*, 2000)

$$p\left(\frac{I}{\langle I\rangle}\right) = \frac{M^M}{\Gamma(M)}\left(\frac{I}{\langle I\rangle}\right)^{M-1}\frac{I}{\langle I\rangle}exp\left(-M\frac{I}{\langle I\rangle}\right), \quad \text{(S2)}$$

where I is the integrated intensity for a single pulse, $\langle \boldsymbol{I}\rangle$ is the average intensity from all pulses and M is the number of modes.

According to the FEL theory (Saldin *et al.*, 2000), the number of modes M is inversely proportional to the normalized dispersion of the intensity distribution

$$M = \frac{\langle I\rangle^2}{\sigma_I^2}, \quad \text{(S3)}$$

where $\boldsymbol{\sigma_I}$ is the standard deviation of the intensity distribution. The results of this analysis were summarized in Table S3.

Table S3 Results of the mode analysis in spatial and spectral domains.

Bunch charge	120 pC			180 pC			200 pC		
Operation mode	SASE	mono	seed	SASE	mono	seed	SASE	mono	seed
M Spatial domain	37.0±4.9 /201.5±62.7 (L)	4.8±0.5 /6.2±0.6 (L)	26.3±3.9	102.6±12.7	3.1±0.1	7.0±1.0	40.2±3.5	4.2±0.4	5.9±0.6 /8.6±0.7 (L)
M Spectral domain	28.3±3.0 /305.1±76.3 (L)	11.7±3.0 /61.2±16.9 (L)	27.1±1.4	43.4±2.8	11.0±2.6	6.3±0.4	64.3±6.2	401.1±296.9	5.7±0.5 /11.8±1.0 (L)

As we can see from Figs. S6- S8 Gamma distribution is observed mostly in monochromatic regime of operation when there are few modes are contributing to the total intensity. In the SASE mode number of modes is large (about 100) and analysis by Gamma distribution is not working. In self-seeding mode pulse distribution is complicated and will need a special analysis.

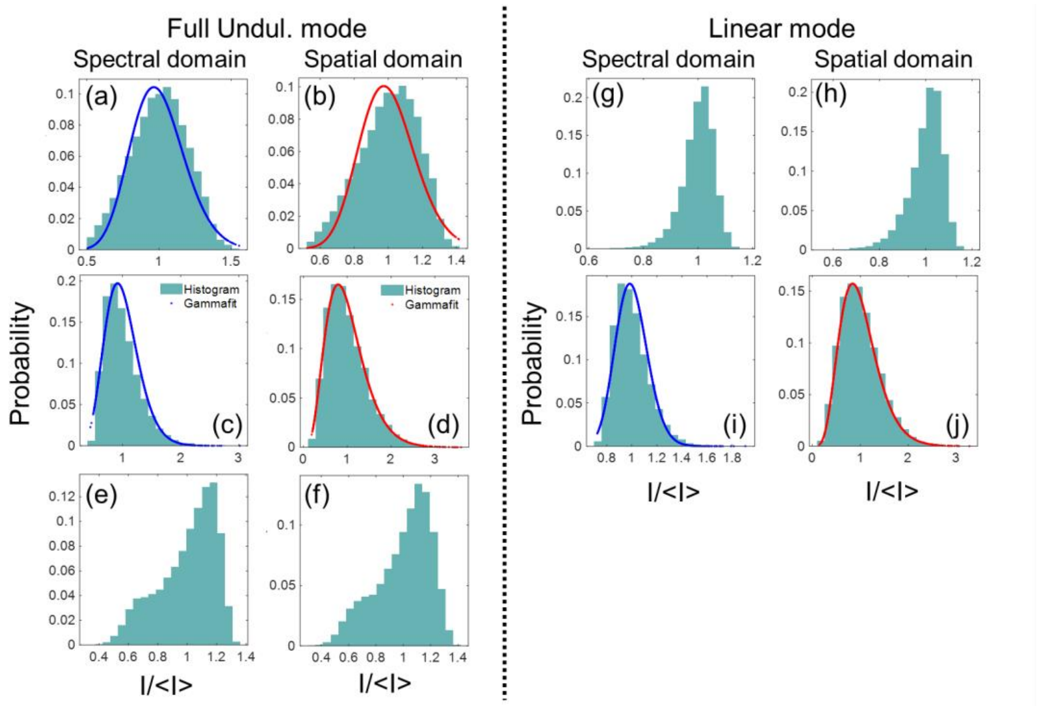

Figure S6 Intensity histograms in the spatial and spectral domains for operation of the PAL XFEL source at the 120 pC bunch charge. Left side: (a,b) SASE mode, (c,d) SASE monochromatic mode, and (e,f) self-seeding mode of operation. Right side, linear mode of operation: (g,h) SASE and (i,j) SASE monochromatic mode. The red and blue lines are the results of Gamma distribution fitting.

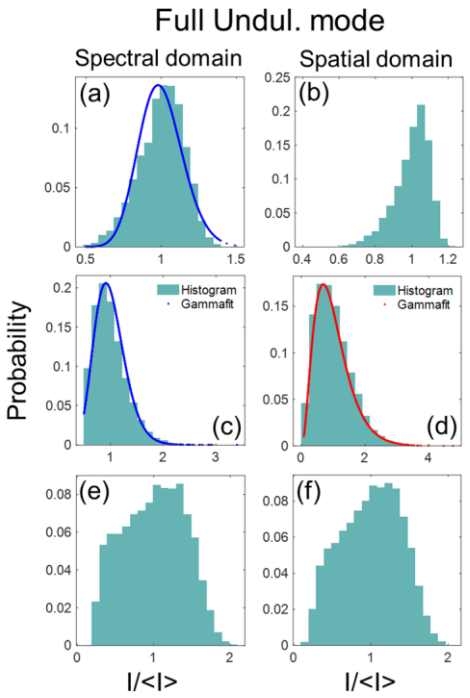

Figure S7 Intensity histograms in the spatial and spectral domains for operation of the PAL XFEL source at the 180 pC bunch charge. (a,b) SASE mode, (c,d) SASE monochromatic mode, and (e,f) self-seeding mode of operation. The red and blue lines are the results of Gamma distribution fitting.

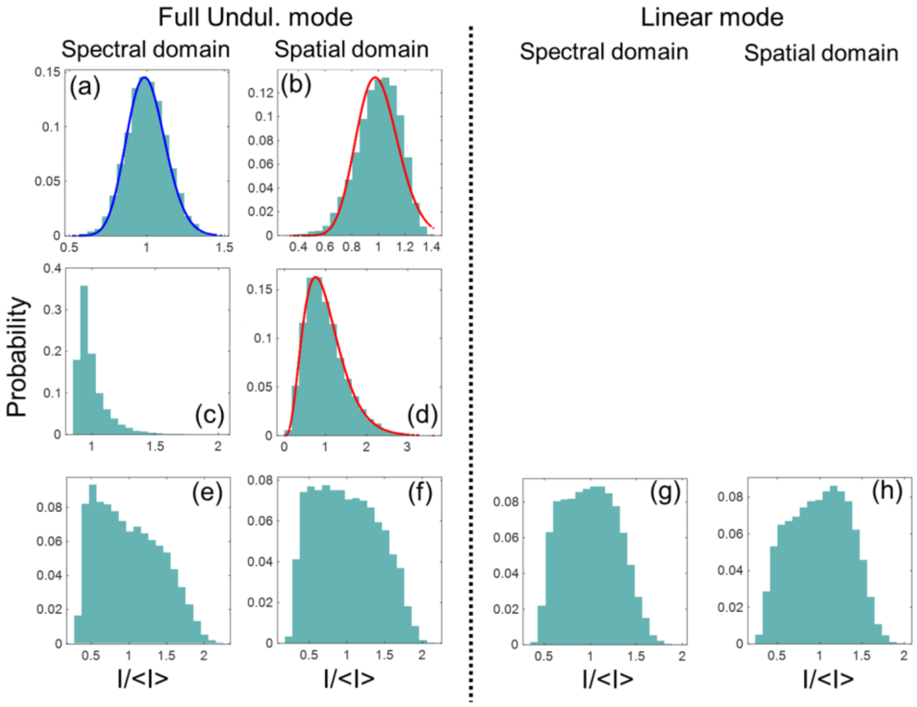

Figure S8 Intensity histograms in the spatial and spectral domains for operation of the PAL XFEL source at the 200 pC bunch charge. Left side: (a,b) SASE mode, (c,d) SASE monochromatic mode, and (e,f) self-seeding mode of operation. Right side, linear mode of operation: (g,h) self-seeding mode of operation in linear regime. The red and blue lines are the results of Gamma distribution fitting.

S6. HBT analysis

S6.1. Spectral analysis

The normalized spectral $g^{(2)}(\omega_1,\omega_2)$-correlation function has the following form

$$g^{(2)}(\omega_1,\omega_2) = \frac{< I(\omega_1 - \omega_0) I(\omega_2 - \omega_0) >}{< I(\omega_1 - \omega_0) >< I(\omega_2 - \omega_0) >}, \quad \text{(S4)}$$

where $\boldsymbol{I(\omega_1)}$ and $\boldsymbol{I(\omega_2)}$ are the intensities of the wave field in spectral representation, $\boldsymbol{\omega_0}$ is the central frequency, and averaging denoted by brackets <…> is performed over a large ensemble of different realizations of the wave field. These spectral correlation functions are presented in Figs. S9-S12 and are discussed in the main part of the paper.

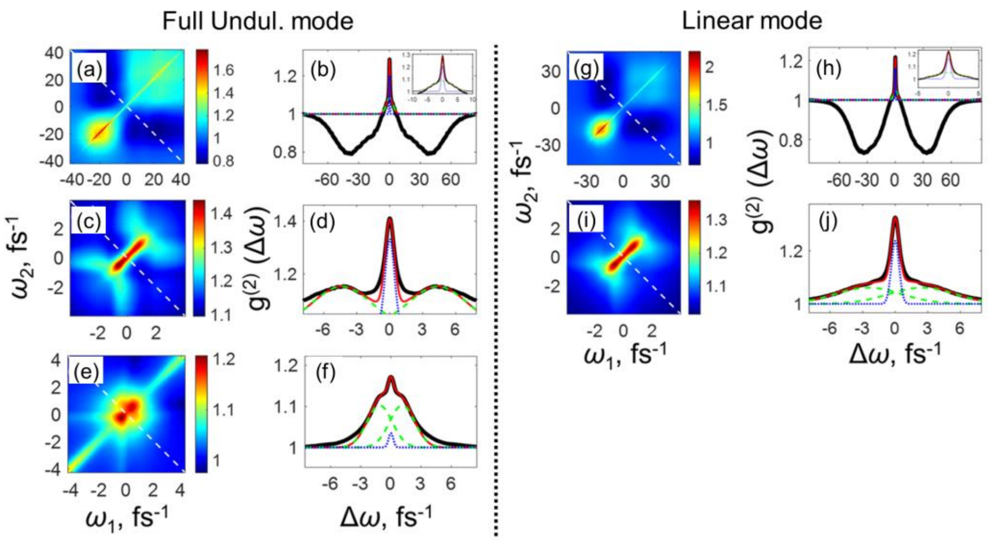

Figure S9 Spectral $g^{(2)}$-function analysis and one-dimensional anti-diagonal profile (along white dash line) for operation of the PAL XFEL source at the 120 pC bunch charge. Left side: (a,b) SASE mode, (c,d) SASE monochromatic mode, and (e,f) self-seeding mode of operation. Right side, linear mode of operation: (g,h) SASE mode, (i,j) SASE monochromatic mode. The inset is enlarged profile at the (b,h). The pulse duration was calculated by blue dash line peak

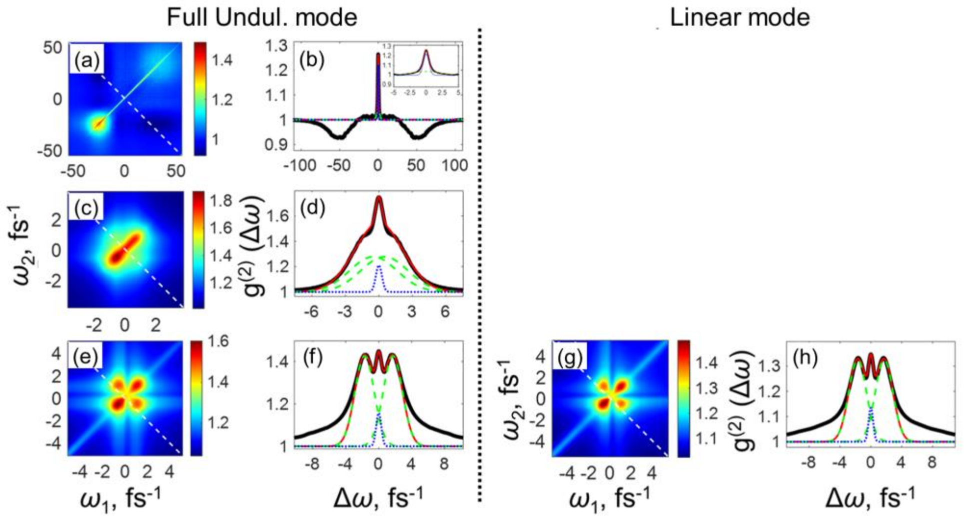

Figure S10 Spectral $g^{(2)}$-function analysis and one-dimensional anti-diagonal profile (along white dash line) for operation of the PAL XFEL source at the 200 pC bunch charge. Left side: (a,b) SASE mode, (c,d) SASE monochromatic mode, and (e,f) self-seeding mode of operation. Right side, linear mode of operation: (g,h) self-seeding mode of operation. The inset is enlarged profile at the (b,h). The pulse duration was calculated by blue dash line peak.

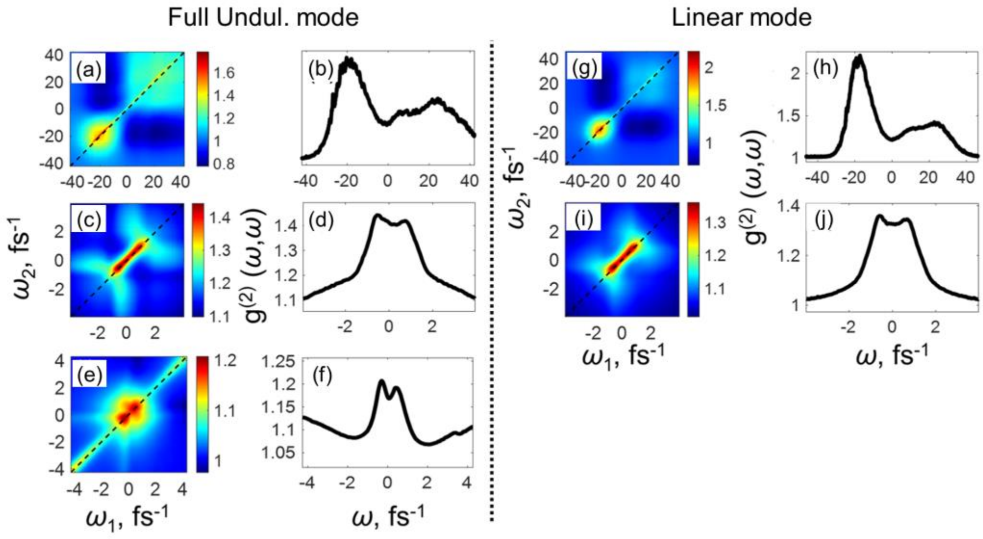

Figure S11 Spectral $g^{(2)}$-function analysis and one-dimensional diagonal profile (along black dash line) for operation of the PAL XFEL source at the 120 pC bunch charge. Left side: (a,b) SASE mode, (c,d) SASE monochromatic mode, and (e,f) self-seeding mode of operation. Right side, linear mode of operation: (g,h) SASE mode, (i,j) SASE monochromatic mode.

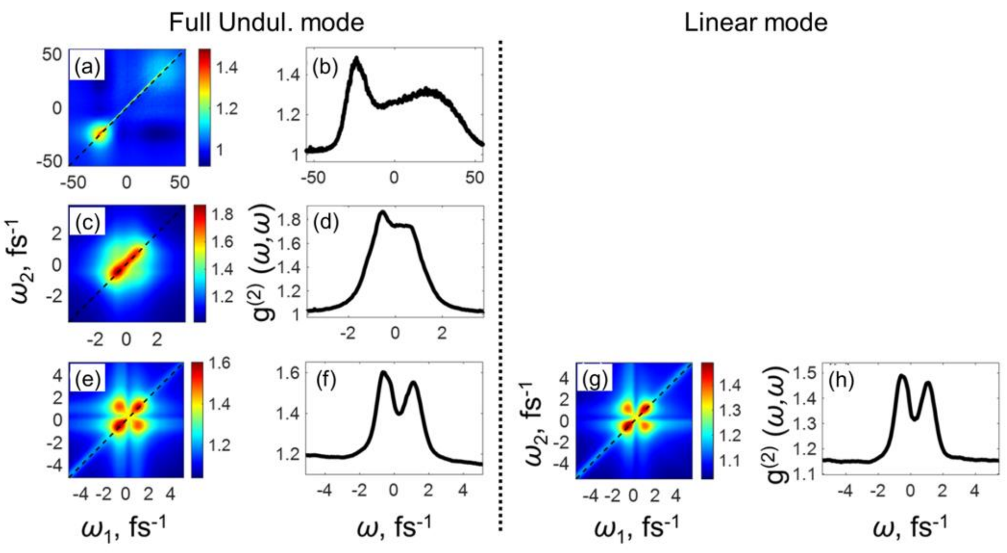

Figure S12 Spectral $g^{(2)}$-function analysis and one-dimensional diagonal profile (along black dash line) for operation of the PAL XFEL source at the 200 pC bunch charge. Left side: (a,b) SASE mode, (c,d) SASE monochromatic mode, and (e,f) self-seeding mode of operation. Right side, linear mode of operation: (g,h) self-seeding mode of operation.

S6.2. Spatial analysis

The normalized spatial second-order correlation function is expressed as

$$g^{(2)}(x_1, x_2) = \frac{\langle I(x_1 - x_0) I(x_2 - x_0)\rangle}{\langle I(x_1 - x_0)\rangle\langle I(x_2 - x_0)\rangle}. \qquad \text{(S5)}$$

Here $I(x) = \int_{-\infty}^{\infty} I(x, y)dy$ is the projected intensity for each pulse in the vertical direction, $I(x_1)$, $I(x_2)$ are the intensities of the wave field at different positions x_1 and x_2, and averaging denoted by brackets <…> is performed over a large ensemble of different realizations of the wave field. Similar to Eq. (S5) expression is valid in the vertical direction. These spatial correlation functions analysed for all operation conditions at PAL XFEL are presented in Figs. S13-S20 and are discussed in the main part of the paper.

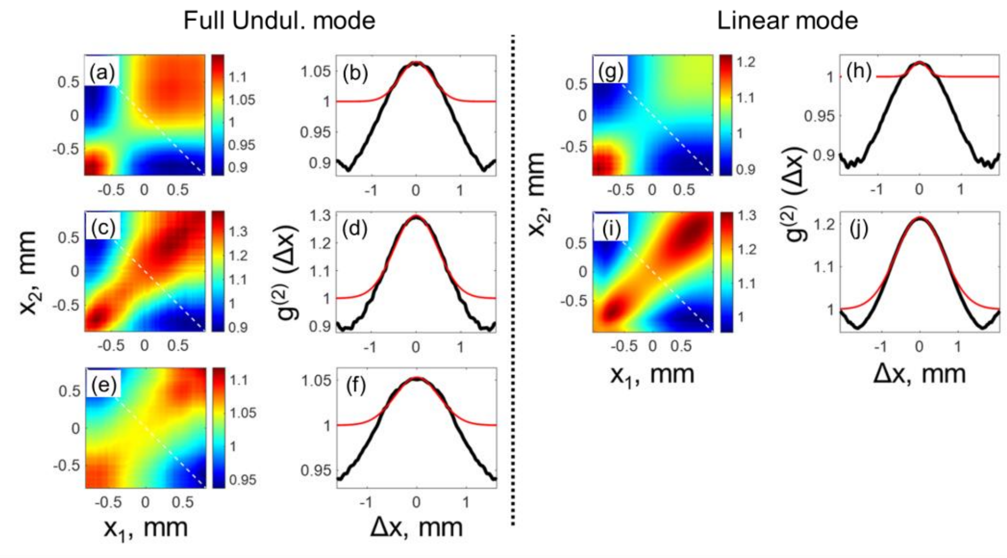

Figure S13 Spatial $g^{(2)}$-function analysis along the horizontal direction and one-dimensional anti-diagonal profile (along white dash line) for operation of the PAL XFEL source at the 120 pC bunch charge. Left side: (a,b) SASE mode, (c,d) SASE monochromatic mode, and (e,f) self-seeding mode of operation. Right side, linear mode of operation: (g,h) SASE mode, (i,j) SASE monochromatic mode. The red lines are the results of Gaussian fitting.

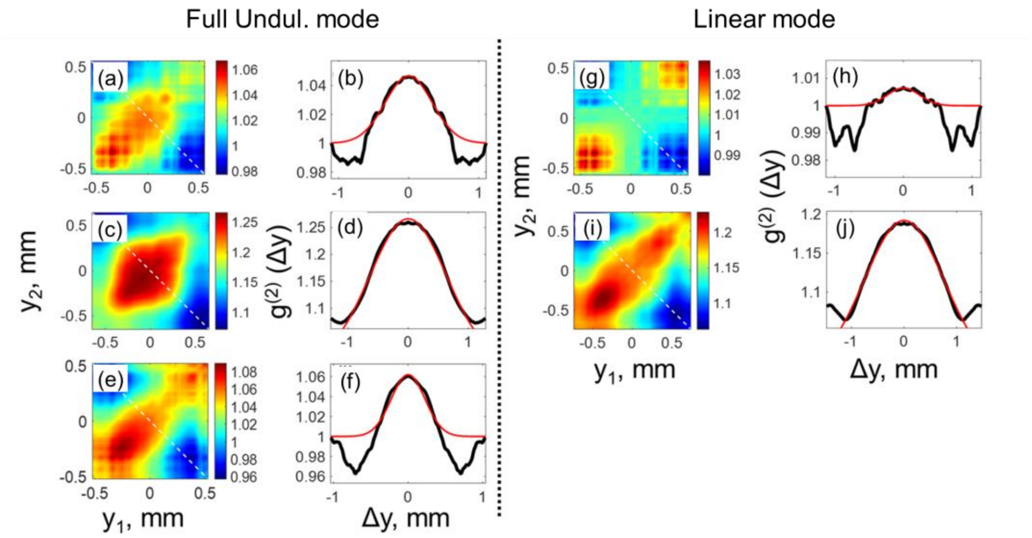

Figure S14 Spatial $g^{(2)}$-function analysis along the vertical direction and one-dimensional anti-diagonal profile (along white dash line) for operation of the PAL XFEL source at the 120 pC bunch charge. Left side: (a,b) SASE mode, (c,d) SASE monochromatic mode, and (e,f) self-seeding mode of operation. Right side, linear mode of operation: (g,h) SASE mode, (i,j) SASE monochromatic mode. The red lines are the results of Gaussian fitting.

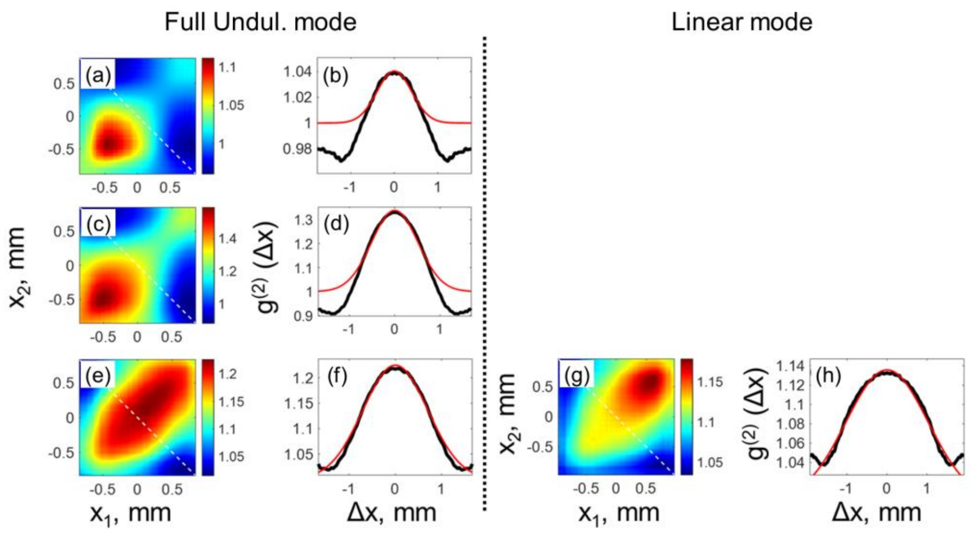

Figure S15 Spatial $g^{(2)}$-function analysis along the horizontal direction and one-dimensional anti-diagonal profile (along white dash line) for operation of the PAL XFEL source at the 200 pC bunch charge. Left side: (a,b) SASE mode, (c,d) SASE monochromatic mode, and (e,f) self-seeding mode of operation. Right side, linear mode of operation: (g,h) self-seeding mode of operation. The red lines are the results of Gaussian fitting.

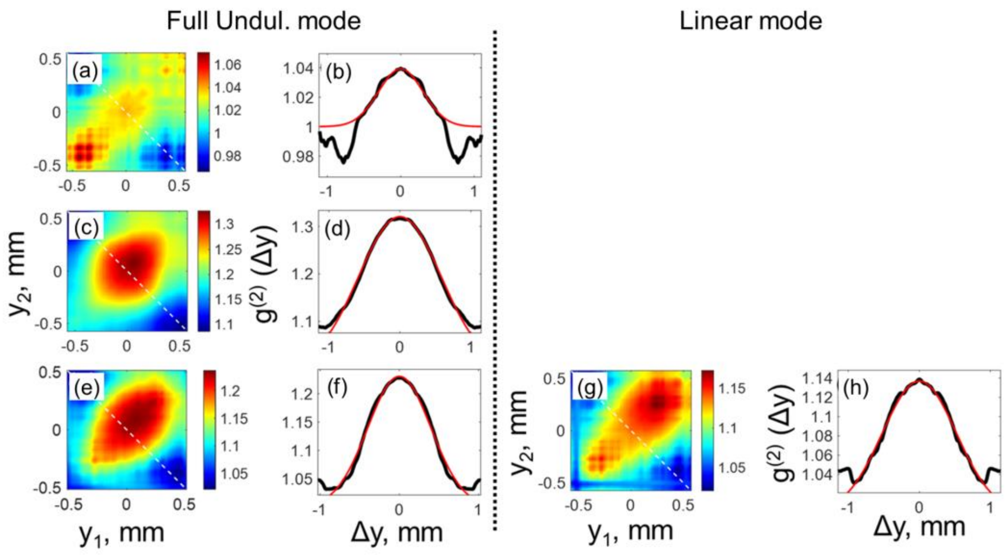

Figure S16 Spatial $g^{(2)}$-function analysis along the vertical direction and one-dimensional anti-diagonal profile (along white dash line) for operation of the PAL XFEL source at the 200 pC bunch charge. Left side: (a,b) SASE mode, (c,d) SASE monochromatic mode, and (e,f) self-seeding mode of operation. Right side, linear mode of operation: (g,h) self-seeding mode of operation. The red lines are the results of Gaussian fitting.

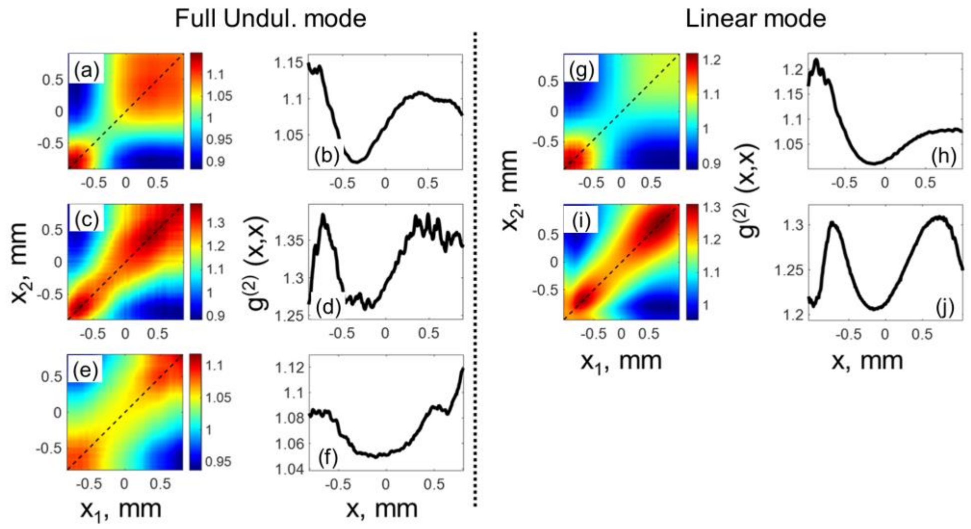

Figure S17 Spatial $g^{(2)}$-function analysis along the horizontal direction and one-dimensional diagonal profile (along black dash line) for operation of the PAL XFEL source at the 120 pC bunch charge. Left side: (a,b) SASE mode, (c,d) SASE monochromatic mode, and (e,f) self-seeding mode of operation. Right side, linear mode of operation: (g,h) SASE mode, (i,j) SASE monochromatic mode.

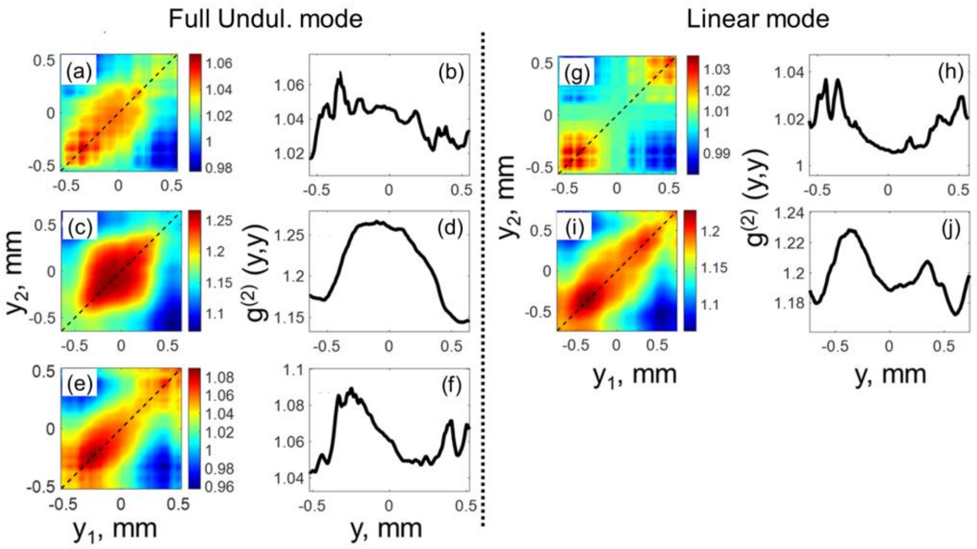

Figure S18 Spatial $g^{(2)}$-function analysis along the vertical direction and one-dimensional diagonal profile (along black dash line) for operation of the PAL XFEL source at the 120 pC bunch charge. Left side: (a,b) SASE mode, (c,d) SASE monochromatic mode, and (e,f) self-seeding mode of operation. Right side, linear mode of operation: (g,h) SASE mode, (i,j) SASE monochromatic mode.

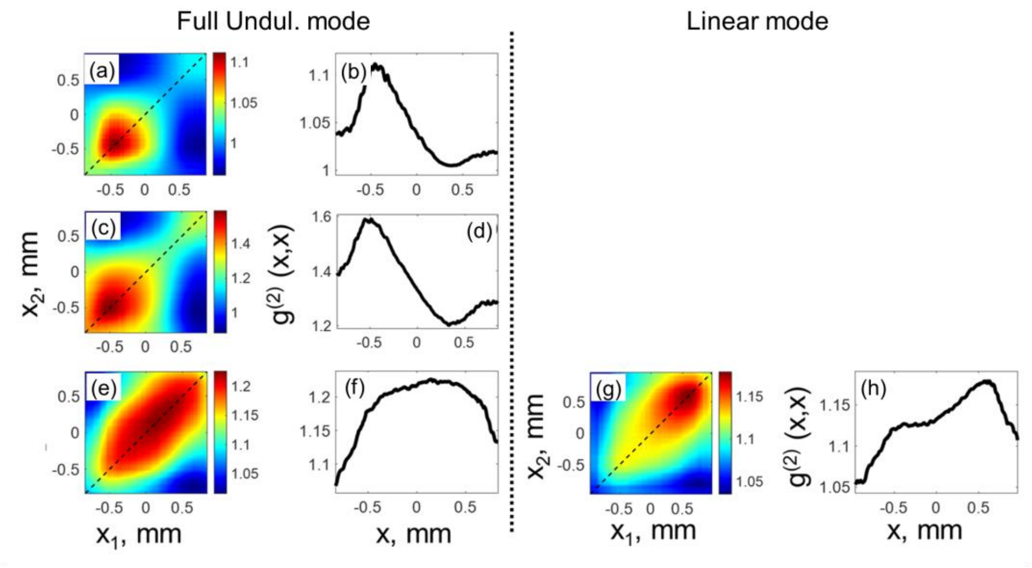

Figure S19 Spatial $g^{(2)}$-function analysis along the horizontal direction and one-dimensional diagonal profile (along black dash line) for operation of the PAL XFEL source at the 200 pC bunch charge. Left side: (a,b) SASE mode, (c,d) SASE monochromatic mode, and (e,f) self-seeding mode of operation. Right side, linear mode of operation: (g,h) self-seeding mode of operation.

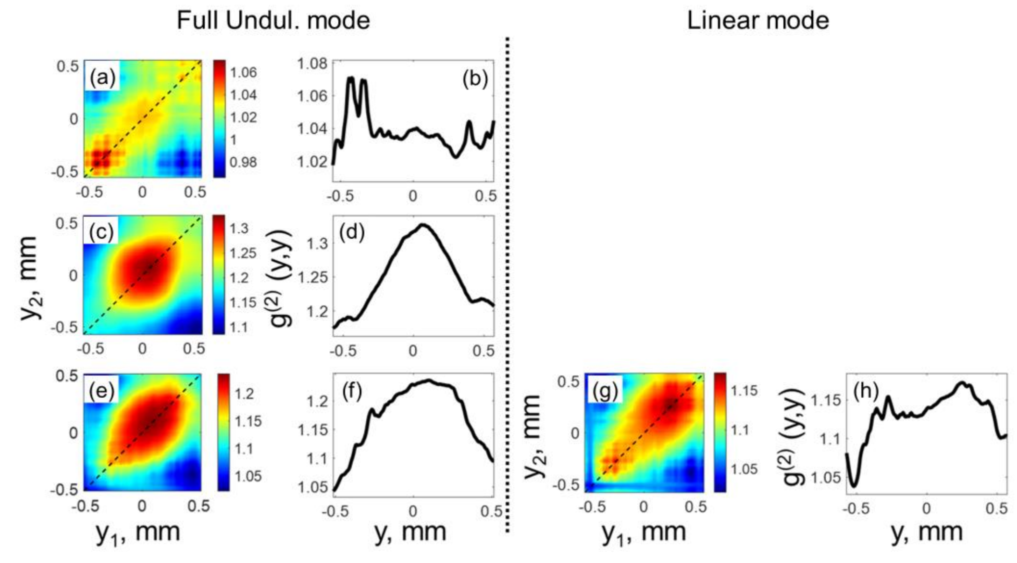

Figure S20 Spatial $g^{(2)}$-function analysis along the vertical direction and one-dimensional diagonal profile (along black dash line) for operation of the PAL XFEL source at the 200 pC bunch charge. Left side: (a,b) SASE mode, (c,d) SASE monochromatic mode, and (e,f) self-seeding mode of operation. Right side, linear mode of operation: (g,h) self-seeding mode of operation.
